# Cost-Effectiveness of Pregabalin, Duloxetine, and Milnacipran vs Amitriptyline for Moderate to Severe Fibromyalgia

**DOI:** 10.1001/jamanetworkopen.2025.57536

**Published:** 2026-02-03

**Authors:** Sarah S. Downen, Hussein M. Farag, Abby Davies, Chijioke M. Okeke, Kenechukwu C. Ben-Umeh, Ismaeel Yunusa, Tewodros Eguale

**Affiliations:** 1 Department of Pharmaceutical Outcomes and Policy, College of Pharmacy, University of Florida, Gainesville; 2 Pharmaceutical Sciences Department, Fakeeh College for Medical Sciences, Jeddah, Saudi Arabia; 3 Department of Pharmaceutical Economics and Policy, Massachusetts College of Pharmacy and Health Sciences, Boston; 4 Department of Clinical Pharmacy and Outcomes Sciences, College of Pharmacy, University of South Carolina, Columbia; 5 Department of Pharmaceutical Health Outcomes and Policy, College of Pharmacy, University of Houston, Houston, Texas; 6 Department of Pharmacotherapy, College of Pharmacy, University of Utah, Salt Lake City; 7 Center for Outcomes Research and Evaluation, University of South Carolina College of Pharmacy, Columbia

## Abstract

**Importance:**

Fibromyalgia (FM) is a chronic pain condition associated with substantial quality-of-life impairments and economic burden. Although multiple pharmacologic options are recommended in clinical guidelines, the relative cost-effectiveness of off-label amitriptyline compared with FDA-approved treatments remains poorly defined due to a scarcity of direct economic comparisons and the use of heterogenous outcome measures in the extant literature.

**Objective:**

To evaluate the cost-effectiveness of pregabalin, duloxetine, and milnacipran compared with amitriptyline in adults with moderate to severe FM.

**Design, Setting, and Participants:**

Decision analytical model using a Markov cohort state transition model to estimate lifetime costs and quality-adjusted life-years (QALYs) for each investigated pharmacologic strategy. Model inputs included treatment-specific transition probabilities, utility values, and direct and indirect costs for each health state, which were derived from published sources. The simulated cohort reflected adults (aged ≥18 years) with moderate to severe FM. Data were analyzed between September 2024 and February 2025.

**Exposure:**

Amitriptyline (25-100 mg), pregabalin (150, 300, 450, and 600 mg), duloxetine (60 and 120 mg), and milnacipran (100 and 200 mg), evaluated from US health care payer and societal perspectives.

**Main Outcomes and Measures:**

Expected lifetime cost, QALYs, incremental cost-effectiveness ratios (ICERs), and incremental net monetary benefit (iNMB) at willingness-to-pay (WTP) thresholds of $50 000, $100 000, and $150 000 per QALY.

**Results:**

The simulated cohort included predominantly women (94.4%), had a mean (SD) age of 48.4 (10.4) years, and was parameterized using demographic distributions derived from published FM populations. From the US health care payer perspective, duloxetine 120 mg was associated with increased QALYs versus amitriptyline at slightly higher cost (ICER, $1536 per QALY), while pregabalin 450 mg was dominated by duloxetine 120 mg. When societal costs were considered, duloxetine 120 mg and pregabalin 450 mg were cost saving relative to amitriptyline. Amitriptyline remained both more effective and less costly than milnacipran, lower doses of pregabalin and duloxetine, and no treatment. At a $100 000 WTP threshold, iNMB for duloxetine 120 mg was $40 375 from the health care payer perspective and $70 063 from the societal perspective; for pregabalin 450 mg, iNMB was $21 211 and $40 190 for the health care payer and societal perspectives, respectively.

**Conclusions and Relevance:**

In this decision analytical model, duloxetine 120 mg was the preferred strategy across perspectives; pregabalin 450 mg was economically favorable relative to amitriptyline only when societal costs were included. Amitriptyline provided greater net benefit than milnacipran and the lower doses of pregabalin and duloxetine. These findings may help inform the selection of value-based treatments for moderate to severe FM.

## Key Points

**Question:**

Among adults with moderate to severe fibromyalgia, how do pregabalin, duloxetine, and milnacipran compare with amitriptyline in terms of cost-effectiveness?

**Findings:**

In this decision analytical model, duloxetine 120 mg and pregabalin 450 mg were more effective and less costly than amitriptyline from the societal perspective. Other evaluated strategies, including lower doses of duloxetine and pregabalin and milnacipran regimes, were dominated by amitriptyline.

**Meaning:**

These findings suggest that whereas most other options provided inferior value, duloxetine 120 mg and pregabalin 450 mg offered greater health benefits at lower costs than amitriptyline in moderate to severe fibromyalgia when societal costs were considered.

## Teaser

This decision analytical model evaluates the cost-effectiveness of pregabalin, duloxetine, and milnacipran compared with amitriptyline in adults with moderate to severe fibromyalgia.

## Introduction

Fibromyalgia (FM) affects an estimated 2% to 6% of US adults, with prevalence varying by diagnostic criteria and surveyed population.^1^ Mean annual US health care costs for patients with FM are nearly 3 times higher than those for age- and sex-matched individuals without the condition ($9573 vs $3291).^2^ Over a 1-year period, patients with FM see their physicians 4 times as often, visit twice as many outpatient facilities, and present to the emergency department 4 times as often as patients without the condition.^2^ Productivity losses associated with an FM diagnosis exceed 1% of the gross domestic product, highlighting the imperative to allocate resources to therapies that deliver the greatest health benefit per dollar.^3^

With an annual estimated global FM market worth $3.6 billion, only 3 pharmacological treatments are currently approved by the US Food and Drug Administration (FDA): pregabalin, approved in 2007; duloxetine, approved in 2008; and milnacipran, approved in 2009.^4,5,6,7^ For pharmacological management, the American College of Rheumatology (ACR) acknowledges the clinical effectiveness of duloxetine, milnacipran, and pregabalin, while including amitriptyline as a recognized option for symptom relief.^8^

International guidelines further recognize amitriptyline’s role in FM care: the German Association of the Scientific Medical Societies recommends low-dose amitriptyline, pregabalin, duloxetine, and milnacipran as first-line pharmacologic treatments.^9^ In contrast, the 2017 guideline from the European League Against Rheumatism conditionally recommends amitriptyline for symptom management but reserves its only strong recommendation for exercise.^10^

Clinical evidence indicates that amitriptyline may offer comparable or superior symptom relief with respect to current FDA approved treatments^11,12,13^: Häuser et al^11^ reported clinically meaningful improvements in pain and sleep with amitriptyline, and a 2022 network meta-analysis of 36 trials showed that amitriptyline was associated with the largest improvements in sleep disturbances, fatigue, and quality of life, whereas duloxetine achieved the greatest reductions in pain and depressive symptoms.^12^

While there is evidence of the effectiveness of amitriptyline, pharmacoeconomic studies comparing the drug with FDA approved FM therapies are limited in scope: Lloyd et al^14^ developed a Markov model examining whether pregabalin was cost-effective compared with other treatments, including amitriptyline, gabapentin, tramadol, duloxetine, and milnacipran. They found that pregabalin 225 mg was cost-saving against duloxetine and milnacipran, but was only cost-saving against amitriptyline when older trials were excluded. Limiting the study’s generalizability, Lloyd et al^14^ did not provide direct comparisons between duloxetine, milnacipran, and amitriptyline. Beard et al^15^ analyzed duloxetine added to standard of care and reported an incremental cost-effectiveness ratio (ICER) of $16 565 per quality-adjusted life-year (QALY) gained. However, to our knowledge, no study has directly compared all treatment options under the same analytic framework to determine which treatment offers the best value for patients with FM.^14,15^ To address this evidentiary gap, we compared the cost-effectiveness of amitriptyline with pregabalin, duloxetine, and milnacipran for moderate to severe FM from a US health care payer and societal perspective.

## Methods

### Study Overview

This study assessed the cost-effectiveness of pharmacologic treatments for FM using a Markov cohort state-transition model. Conducted from both a US health care payer and a US societal perspective, the analysis adhered to the Second Panel on Cost-effectiveness in Health and Medicine guidelines. Because the study population was simulated based on published data, the study was exempt from institutional review board approval and the need for informed consent, in accordance with 45 CFR §46. Results were reported in accordance with the Consolidated Health Economic Evaluation Reporting Standards (CHEERS) reporting guideline.^16,17,18,19^

### Model Structure and Assumptions

**Figure 1.**
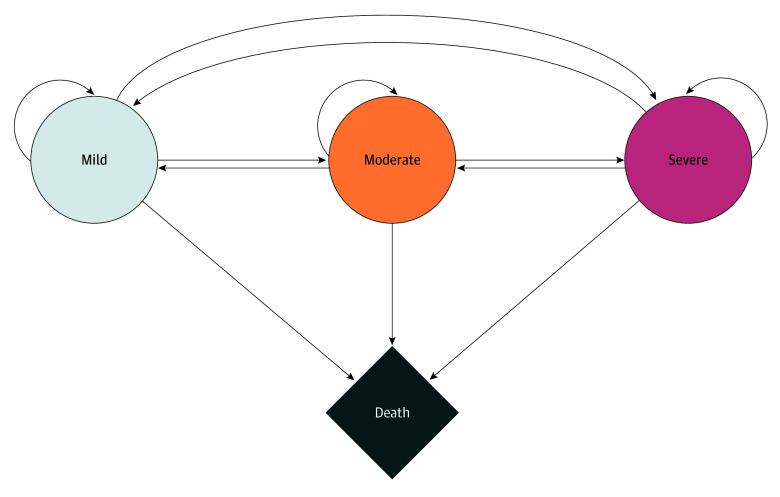
Model Structure of Health-State Transitions in Fibromylagia The arrows represent a possible transition from one state to another, either unilateral (to death state), bilateral (between mild, moderate, and severe states), or to remain in the same state.

The Markov model included 4 health states: mild, moderate, severe, and death. We adapted this structure from previous models designed by Tarride et al^20^ and Ornelas et al,^21^ respectively. Individuals entered the cohort in either the moderate or severe states, consistent with the health status of patients at the time of initiation of pharmacotherapy in FM cohort studies and clinical practice.^14,15,21^ Health states were defined using an 11-point pain scale: mild (0-3), moderate (4-6), and severe (7-10).^22,23,24,25,26,27,28^ This classification aligns with validated cutoffs widely used in FM clinical trials and routine care. During each annual cycle, individuals were able to remain in their current state, transition to a higher or lower pain state based on treatment effects and disease progression, or transition to death from any health state. (Figure 1). The Markov model was extended until no additional life-years or costs accrued, ensuring full representation of a lifetime horizon. This time horizon aligns with recommended guidelines for chronic and progressive conditions, where short-term modeling may underestimate the cumulative burden of disease and treatment.^17^

### Population and Interventions

The simulated cohort included adults (aged ≥18 years) diagnosed with FM who were predominantly women, parameterized using demographic distributions derived from published FM populations.^29,30,31^ Inclusion criteria were aligned with the diagnostic standards most widely accepted in clinical and research settings: American College of Rheumatology (ACR) 1990, ACR 2010, Yunus, and Smyth criteria.^32,33,34,35,36^ These distributions were used to initialize the simulated population at baseline, ensuring representativeness of the FM population commonly encountered in clinical practice eligible for pharmacologic management.

The analysis included 4 pharmacological interventions consistent with international guidelines and clinical prescribing patterns: off-label amitriptyline and the FDA-approved therapies pregabalin (150, 300, 450, and 600 mg), duloxetine (60 and 120 mg), and milnacipran (100 and 200 mg).^10,37,38,39^ The selected doses reflect those commonly used in routine care and which were assessed in pivotal randomized clinical trials (RCTs).^40,41,42,43,44,45,46,47,48,49,50,51^

### Model Inputs

#### Transition Probabilities

The probabilities of transitioning between health states were derived from a Bayesian network meta-analysis of 36 RCTs, allowing integration of both direct and indirect comparative evidence across all interventions.^52^ Background all-cause mortality was incorporated using age- and sex-specific rates from the 2021 National Vital Statistics System US life tables.^53^

#### Costs

All cost estimates were standardized to 2024 US dollars using the medical component of the consumer price index.^54,55^ Drug acquisition costs for each intervention were obtained from the wholesale acquisition cost reported in the Red Book.^56^ Estimates for other direct costs, including hospitalizations, outpatient visits, emergency care, diagnostic testing, and out-of-pocket expenditures, were derived from published studies on the economic burden of FM.^57^ Indirect costs, such as productivity losses, disability-related work absences, and unpaid caregiving, were included when analyses were conducted from the US societal perspective.^57^ These societal components were operationalized as 3-month indirect cost estimates that were mapped to the model health states (mild, moderate, and severe).^57^ The US health care payer perspective incorporated only direct medical and nonmedical costs.^26,57^

#### Utilities

Utility values for mild, moderate, and severe FM pain were derived from published studies using validated health-related quality-of-life instruments, including the EuroQol 5-Dimension (EQ-5D), Fibromyalgia Impact Questionnaire, Multidimensional Assessment of Fatigue, Medical Outcomes Study Sleep Scale, and Hospital Anxiety and Depression Scale.^25,58,59,60,61,62,63^ Full details of the input parameters, including the utility values, are provided in eTables 1 through 4 in Supplement 1.

### Statistical Analysis

We estimated expected lifetime costs and QALYs for each pharmacologic strategy, applying a 3% annual discount rate to both outcomes. Analyses were conducted from the US health care payer and US societal perspectives, with perspective-specific cost components included accordingly.^17^ Strategies were ordered by increasing cost. Any strategy that resulted in higher costs and lower QALYs compared with another was classified as strongly dominated and excluded since it cannot provide a greater health benefit or economic value than at least 1 alternative. Among options that were not strongly dominated, ICERs were calculated for each adjacent pair. If a treatment had an ICER higher than that of a more effective and more costly option, it was considered extendedly dominated (eg, weak dominance on the efficiency frontier) and excluded. This sequential process ensured that all retained options represented efficient uses of resources, where a higher cost was justified by greater effectiveness.^64,65^ On the basis of this process, remaining strategies form the efficient frontier, representing the set of nondominated options that provide the greatest health benefit for their associated cost. Interventions located on the efficient frontier are those that would be considered relevant for value-based decision-making because no alternative offers more QALYs at the same or lower cost. Strategies not on this frontier are considered economically inferior since they either cost more for the same benefit or offer less benefit for a comparable cost.

For all interventions, incremental net monetary benefit (iNMB) was calculated at willingness-to-pay thresholds (WTP) of $50 000, $100 000, and $150 000 per QALY. iNMB for each strategy was defined as the difference in net monetary benefit between that strategy and the reference, calculated as (WTP × incremental QALYs) – incremental cost.^66,67^ This quantity represents the net value of the health gain relative to the added expenditure. When iNMB is positive, the evaluated option delivers more value than it costs at the specified WTP. A negative result indicates that the improvement in health does not justify the increase in spending. An iNMB of 0 implies equal efficiency relative to the comparator. This formulation avoids interpretive limitations of the ICER, particularly when comparisons involve dominated or closely aligned alternatives. It also reflects the underlying principles of decision theory by representing the negative of the incremental loss, thereby enabling coherent value-based comparisons across all options.^64,65^ A half-cycle correction was applied to both costs and health outcomes to account for the fact that transitions between health states may occur at any time during the annual cycle. This approach adjusts for midcycle timing of events and aligns with recommended good practice guidelines for state-transition models.^68^

Robustness of the findings was evaluated using both deterministic and probabilistic sensitivity analyses (PSA). Deterministic analysis consisted of multiple 1-way sensitivity analyses in which each parameter was varied independently across its plausible range while holding all others constant. This approach identified the individual model inputs that were most strongly associated with estimates of cost, QALYs, and iNMB. The PSA was conducted using 1000 second-order Monte Carlo simulations in which all parameters were varied simultaneously according to their base-case probability distributions to reflect joint uncertainty in the model inputs.

Beta distributions were assigned to transition probabilities.^69,70^ Gamma distributions were selected for cost parameters to accommodate the right-skewed nature of cost data and variability across severity levels in FM. Lognormal distributions were used for utility values, reflecting the empirical distribution of utilities in chronic pain and accounting for the observed lower bounds in severe states and near-death.^71,72^ The use of lognormal rather than beta distributions for utilities follows methodological recommendations for situations where utility values can approach 0, as is often observed in severe FM and older age.^73^ Data were analyzed between September 2024 and February 2025. All analyses were performed using Excel 2021 (Microsoft) and the hēRo3 (Avalere Health) web-based, open-source health economic modeling platform.^74^ All results are reported as expected mean per patient.

## Results

### Base Case

**Figure 2.**
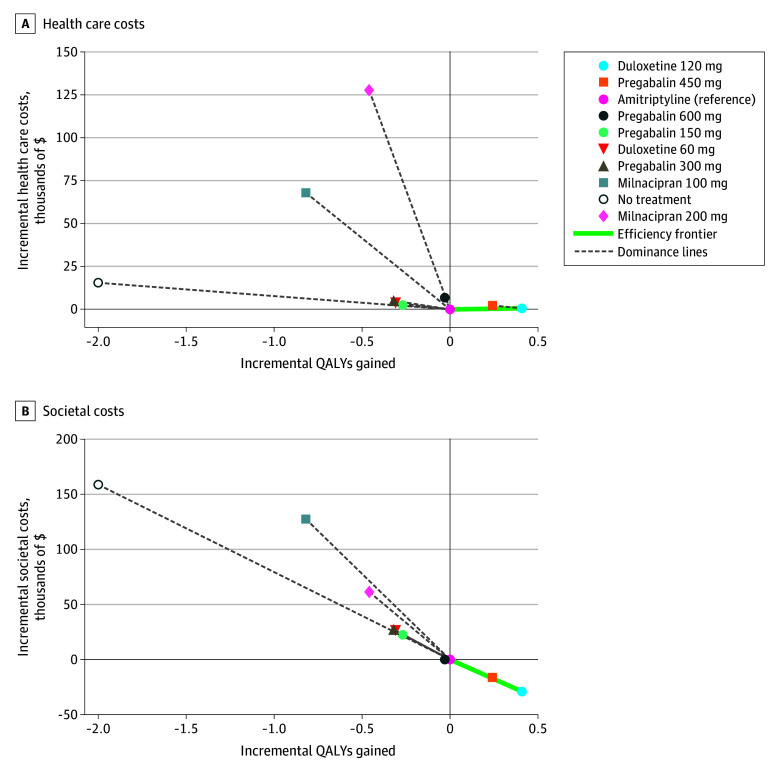
Incremental Cost-Effectiveness Frontier Each point represents a treatment strategy, plotted by its incremental cost (y-axis) and incremental QALYs (x-axis) relative to amitriptyline (reference, shown at the origin). The efficient frontier (green line) connects nondominated strategies representing treatments where any additional cost is justified by additional health benefit. Strategies to the right of and below another strategy offer better value (more QALYs, lower cost). Strategies above and to the left of the frontier are dominated, meaning a more effective and less costly alternative exists. Dashed gray lines connect dominated strategies to the frontier for visual reference.

The simulated cohort included adults (aged ≥18 years) diagnosed with FM who were predominantly women (94.4%) with a mean (SD) age of 48.4 (10.4) years. From the US health care payer perspective, only amitriptyline and duloxetine 120 mg appeared on the efficient frontier (Figure 2A). Duloxetine 120 mg yielded greater health benefits than amitriptyline (10.40 vs 9.99 QALYs) at a slightly higher cost ($115 770 vs $115 145), resulting in an ICER of $1536 per QALY. All other interventions were dominated, including all doses of pregabalin, duloxetine 60 mg, both milnacipran regimens, and no treatment (Table 1). Notably, pregabalin 450 mg had both a higher cost ($117 434 vs $115 770) and lower effectiveness (10.23 vs 10.40 QALYs) than duloxetine 120 mg. The resulting iNMB for duloxetine 120 mg was $40 075 over amitriptyline at a WTP threshold of $100 000 per QALY.

**Table 1.** Base Case: Cost-Effectiveness of Pregabalin, Duloxetine, and Milnacipran Compared With Amitriptyline, US Health Care Payer Perspective

Treatment	Expected Cost, US$	Expected QALY Gained	ICER, US$/QALY^b^	iNMB^a^		
				$At $50 000/QALY	$At $100 000/QALY	$At $150 000/QALY
Amitriptyline	115 145	9.994	Reference	Reference	Reference	Reference
Duloxetine 120 mg	115 770	10.401	1536	19 725	40 075	60 425
Pregabalin 450 mg	117 434	10.233	Dominated	9709	21 209	32 709
Pregabalin 150 mg	117 676	9.721	Dominated	−15 029	−29 529	−44 029
Duloxetine 60 mg	118 950	9.683	Dominated	−19 303	−34 303	−49 303
Pregabalin 300 mg	120 241	9.676	Dominated	−21 094	−37 094	−53 094
Pregabalin 600 mg	122 006	9.957	Dominated	−8359	−10 359	−12 359
No treatment	130 669	7.990	Dominated	−115 522	−215 522	−315 522
Milnacipran 100 mg	183 138	9.170	Dominated	−108 991	−149 991	−190 991
Milnacipran 200 mg	242 864	9.532	Dominated	−150 717	−173 717	−196 717

Abbreviations: ICER, incremental cost-effectiveness ratio; iNMB, incremental net monetary benefit; QALY, quality-adjusted life-year.

^a^ Incremental net monetary benefit calculated as (incremental QALYs × willingness-to-pay threshold) − incremental cost, rounded to nearest dollar.

^b^ All comparisons are related to amitriptyline as reference treatment. Dominated indicates higher cost and lower effectiveness.

The US societal analysis revealed duloxetine 120 mg as the optimal strategy, dominating all comparators with the highest QALYs (10.40) at the lowest lifetime cost ($712 910). Although amitriptyline and pregabalin 450 mg also appeared on the efficient frontier (Figure 2B), they were both dominated by duloxetine 120 mg. Pregabalin 450 mg, in turn, was associated with lower lifetime costs and higher QALYs than amitriptyline (Table 2). At a $100 000 WTP threshold, the iNMB was $70 063 for duloxetine 120 mg and $40 190 for pregabalin 450 mg, each relative to amitriptyline.

**Table 2.** Base Case: Cost-Effectiveness of Pregabalin, Duloxetine, and Milnacipran Compared With Amitriptyline, US Societal Perspective

Treatment	Expected cost, US$	Expected QALY gained	ICER, US$/QALY^b^	iNMB^a^		
				$At $50 000/QALY	$At $100 000/QALY	$At $150 000/QALY
Duloxetine 120 mg	712 910	10.40	Cost-saving, more effective	49 563	70 063	90 563
Pregabalin 450 mg	725 782	10.23	Cost-saving, more effective	28 190	40 190	52 190
Amitriptyline	741 972	9.99	Reference	Reference	Reference	Reference
Pregabalin 600 mg	742 000	9.96	Dominated	−1528	−3028	−4528
Pregabalin 150 mg	764 605	9.72	Dominated	−36 133	−49 633	−63 133
Duloxetine 60 mg	768 317	9.68	Dominated	−41 845	−57 345	−72 845
Pregabalin 300 mg	769 317	9.67	Dominated	−43 345	−59 345	−75 345
Milnacipran 200 mg	803 430	9.53	Dominated	−84 458	−107 458	−130 458
Milnacipran 100 mg	869 546	9.17	Dominated	−168 574	−209 574	−250 574
No treatment	900 789	7.99	Dominated	−258 817	−358 817	−458 817

Abbreviations: ICER, incremental cost-effectiveness ratio; iNMB, incremental net monetary benefit; QALY, quality-adjusted life-year.

^a^ Incremental net monetary benefit calculated as (incremental QALYs × willingness-to-pay threshold) − incremental cost, rounded to nearest dollar.

^b^ All comparisons are related to amitriptyline as reference treatment. Cost-saving, more effective indicates lower cost and higher effectiveness; dominated indicates higher cost and lower effectiveness; extendedly dominated indicates dominated by linear combination of other interventions.

### Sensitivity Analyses

Deterministic 1-way sensitivity analyses identified the utility assigned to moderate FM and the transition probability from severe to moderate as the most influential parameters. These variables contributed to the greatest variation in iNMB estimates, especially for strategies involving amitriptyline (eFigures 2 and 3 in Supplement 1).

**Figure 3.**
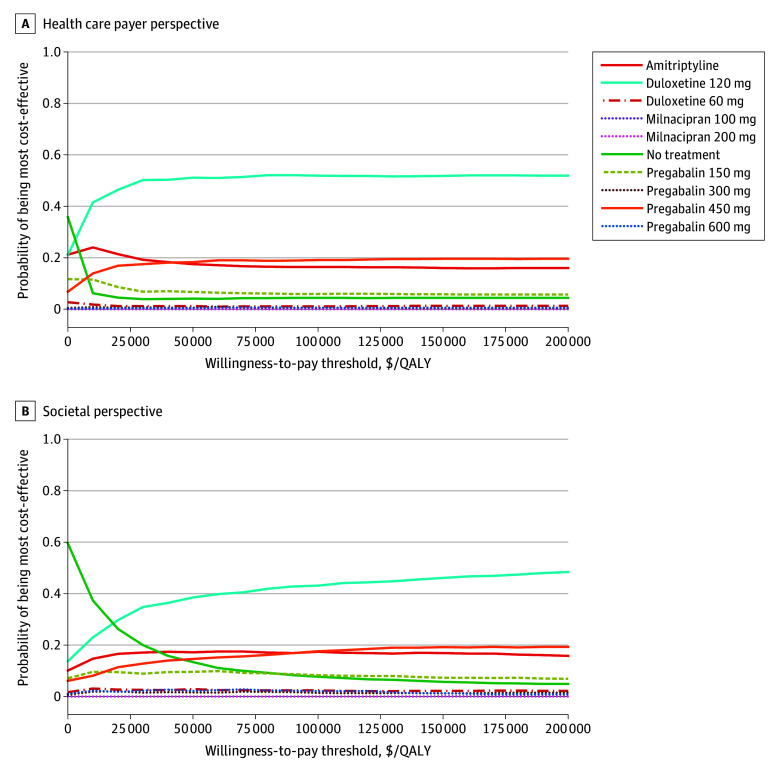
Cost-Effectiveness Acceptability Curve for the Included Treatments for Fibromyalgia Curves show the probability that each treatment for fibromyalgia is the most cost-effective at different willingness-to-pay thresholds.

Figure 3A represents the PSA results from the US health care payer perspective, showing that duloxetine 120 mg had the highest probability of being cost-effective across most of the WTP ranges while not exceeding 55% at any threshold. Amitriptyline was most likely to be cost-effective at low WTP values, but its probability declined sharply above $30 000 per QALY and remained below 20% at higher thresholds. Figure 3B shows a similar pattern from the US societal perspective where duloxetine 120 mg had the highest probability of reaching cost-effectiveness while approaching only 45% at $100 000 per QALY. For both perspectives, pregabalin 450 mg maintained a probability between 15% and 20% at higher thresholds but never approached the probability of duloxetine 120 mg. No strategy exceeded a 60% probability of being cost-effective at any point, indicating that the ranking is subject to considerable uncertainty, even for the leading treatments.

## Discussion

This cost-effectiveness analysis reveals that duloxetine 120 mg and pregabalin 450 mg achieve lower lifetime costs while generating more QALYs than amitriptyline when a US health care payer perspective is applied. These results suggest that improved symptom control reduces downstream health care utilization and offsets the higher acquisition costs of pregabalin and duloxetine compared with generic amitriptyline.

Amitriptyline produced more QALYs at a lower lifetime cost than 6 alternative strategies. These included pregabalin at 150 mg, 300 mg, and 600 mg, duloxetine 60 mg, and both milnacipran regimens. This pattern persisted across both US health care payer and societal perspectives and suggests that these treatments increase expenditures without improving health outcomes regardless of the cost perspective applied

The cost savings associated with duloxetine 120 mg and pregabalin 450 mg became more pronounced from a US societal perspective. This finding reflects the substantial impact of FM on work productivity and caregiver burden. Because the condition disproportionately affects working-age adults, the societal perspective captures a broader portion of the disease burden than the health care sector perspective. Therapies that demonstrate clinical benefit may therefore yield improved economic efficiency when indirect costs are included. These findings suggest strong economic incentives for employers and disability insurers to support access to treatments that effectively manage FM symptoms.

The PSA introduces important nuances to these findings. Duloxetine 120 mg achieved the highest probability of being cost-effective but never exceeded 55% at any WTP threshold. This uncertainty stems primarily from variability in utility values assigned to moderate and severe pain states, which was the prime contributor to the variance in the deterministic sensitivity analyses. Although duloxetine 120 mg represents the optimal choice, the substantial uncertainty suggests other treatments might offer a more favorable balance of costs and QALYs in nearly half of the plausible scenarios.

These results both align with and diverge from previous economic evaluations. Supporting our results, Lloyd et al^14^ found pregabalin to be cost-effective, although their model structure differed substantially from ours and they excluded older amitriptyline trials from some comparisons. Beard et al^15^ reported an ICER of $16 565 per QALY for duloxetine when added to standard care, suggesting good value but not the cost-saving profile identified in the present analysis. These differences likely reflect our direct comparison between active treatments, inclusion of the 120 mg duloxetine dose, and use of a network meta-analysis to synthesize all available evidence.

The dose-response association for duloxetine merits particular attention. While duloxetine 60 mg cost more and produced fewer QALYs than amitriptyline, doubling the dose to 120 mg transformed duloxetine into a cost-saving intervention. This finding highlights how dose optimization can fundamentally alter cost-effectiveness associations. For US health care payers, these findings offer opportunities to improve economic efficiency through evidence-based formulary design. Similarly, dose restrictions that prevent optimization to duloxetine 120 mg may perpetuate inadequate treatment while increasing long-term costs. Rational formulary design should facilitate access to treatments demonstrating favorable cost-effectiveness while reassessing continued coverage of options that yield fewer QALYs at higher costs.

The sustained market dominance of treatments showing lower value across all analyses points to potential market failures in pharmaceutical pricing and coverage decisions.^75^ This formulary placement may reflect factors beyond clinical and economic evidence, including marketing influences, formulary inertia, and the complex relationships between pharmaceutical manufacturers and pharmacy benefit managers.

This analysis demonstrates that optimal FM treatment from an economic perspective differs from current prescribing patterns and formulary designs. Future research should address key uncertainties identified in this analysis. The substantial parameter uncertainty surrounding moderate and severe FM suggests a need for targeted studies in these populations. Restricting trial enrollment to patients with moderate to severe symptoms could improve precision in effectiveness estimates and reduce uncertainty in economic evaluations. Development of FM-specific utility measures that capture the condition’s multidimensional impact would enable a more accurate valuation of treatment benefits. Clinical evidence examining treatment sequences and long-term outcomes could inform more realistic model structures that better reflect clinical practice patterns.

### Limitations

Several limitations should be considered when interpreting our findings. The Markov model in this study represents clinical care as transitions among defined health states. In clinical practice, management of FM may involve dose adjustments, combination therapy, or sequential medication trials that are not fully reflected in this framework. Although the utility values used in this analysis were derived from validated quality-of-life instruments that capture multiple health domains, including function, mood, and cognition, they may not fully represent the full extent of FM’s burden as experienced by patients. Generic measures such as the EQ-5D are constrained by the domains they include and the valuation methods used. As a result, aspects such as cognitive dysfunction, fatigue, and subtle psychological changes may be insufficiently reflected in the utilities assigned to our model’s health states.

Cost estimates derived from published sources might not fully account for variation in local practice patterns or regional health care prices, contributing additional uncertainty to our results. This analysis does not address patient-level heterogeneity in treatment response, a recognized challenge in economic evaluations of conditions with diverse clinical presentations. While the results provide policy-relevant estimates at the population level, individual patients may have different benefit-risk profiles or contraindications based on their characteristics and comorbidities.

## Conclusions

In this model-based cost-effectiveness analysis of adults with moderate to severe fibromyalgia, duloxetine 120 mg provided greater QALYs than amitriptyline from both a US health care payer and societal perspective, with lower lifetime costs observed when indirect costs were included. Under the societal perspective, pregabalin 450 mg also demonstrated lower costs and greater QALYs than amitriptyline, although it was dominated by duloxetine 120 mg. Other treatments, including lower doses of pregabalin and duloxetine and both milnacipran regimens, were less effective and more expensive than amitriptyline. These findings suggest that duloxetine 120 mg offers the greatest economic value for this patient population while highlighting the importance of accounting for societal costs in fibromyalgia treatment decisions. Clinical decisions should additionally consider adverse event profiles, patient comorbidities, and expected variability in treatment response.

## Notes

Supplement 1. eTable 1. Transition Matrices eTable 2. Direct and Indirect Costs eTable 3. Utilities eTable 4. 2021 US Life Tables eFigure 1. Markov Traces of the Included Treatments for Fibromyalgia eFigure 2. Tornado Diagram of Deterministic Sensitivity Analyses, US Health Care Payer Perspective eFigure 3. Tornado Diagram of Deterministic Sensitivity Analyses, US Societal Perspective eReferences.

Supplement 2. Data Sharing Statement
