# Supplementary material for: Cost-Effectiveness of Pregabalin, Duloxetine, and Milnacipran vs Amitriptyline for Moderate to Severe Fibromyalgia
Source: JAMA Netw Open. 2026 Feb 3;9(2):e2557536. doi: 10.1001/jamanetworkopen.2025.57536 (PMC12869342; doi:10.1001/jamanetworkopen.2025.57536)
Supplement: Supplement 1. — eTable 1. Transition Matrices eTable 2. Direct and Indirect Costs eTable 3. Utilities eTable 4. 2021 US Life Tables eFigure 1. Markov Traces of the Included Treatments for Fibromyalgia eFigure 2. Tornado Diagram of Deterministic Sensitivity Analyses, US Health Care Payer Perspective eFigure 3. Tornado Diagram of Deterministic Sensitivity Analyses, US Societal Perspective eReferences. [file jamanetwopen-e2557536-s001.pdf]

Supplemental Online Content

Downen SS, Farag HM, Davies A, et al. Cost-effectiveness of pregabalin, duloxetine, and milnacipran compared with amitriptyline for fibromyalgia. *JAMA Netw Open*. 2026;9(2):e2557536. doi:10.1001/jamanetworkopen.2025.57536

eTable 1. Transition Matrices
eTable 2. Direct and Indirect Costs
eTable 3. Utilities
eTable 4. 2021 US Life Tables
eFigure 1. Markov Traces of the Included Treatments for Fibromyalgia
eFigure 2. Tornado Diagram of Deterministic Sensitivity Analyses, US Health Care Payer Perspective
eFigure 3. Tornado Diagram of Deterministic Sensitivity Analyses, US Societal Perspective
eReferences

This supplemental material has been provided by the authors to give readers additional information about their work.

© 2026 Downen SS et al. *JAMA Network Open*.

eTable 1. Transition Matrices (Transition Probabilities From Shaefer et al.)[1]

No Treatment				Amitriptyline (SMD= -0.27)			
From	**To Mild**	**To Moderate**	**To Severe**	From	**To Mild**	**To Moderate**	**To Severe**
**Mild**	0.571	0.286	0.143	**Mild**	0.648	0.325	0.027
**Moderate**	0.143	0.490	0.37	**Moderate**	0.182	0.622	0.196
**Severe**	0.008	0.333	0.659	**Severe**	0.010	0.430	0.560
**Pregabalin 300mg (SMD= -0.23)**				**Pregabalin 150mg (SMD=-0.23)**			
From	**To Mild**	**To Moderate**	**To Severe**	From	**To Mild**	**To Moderate**	**To Severe**
**Mild**	0.637	0.319	0.044	**Mild**	0.637	0.319	0.044
**Moderate**	0.176	0.603	0.221	**Moderate**	0.176	0.603	0.221
**Severe**	0.010	0.409	0.581	**Severe**	0.010	0.409	0.581
**Pregabalin 450mg (SMD=-0.30)**				**Pregabalin 600mg (SMD= -0.27)**			
From	**To Mild**	**To Moderate**	**To Severe**	From	**To Mild**	**To Moderate**	**To Severe**
**Mild**	0.657	0.329	0.014	**Mild**	0.648	0.325	0.027
**Moderate**	0.186	0.637	0.177	**Moderate**	0.182	0.622	0.196
**Severe**	0.010	0.433	0.557	**Severe**	0.010	0.430	0.560
**Duloxetine 60mg (SMD= -0.24)**				**Duloxetine 120mg (SMD= -0.33)**			
From	**To Mild**	**To Moderate**	**To Severe**	From	**To Mild**	**To Moderate**	**To Severe**
**Mild**	0.640	0.320	0.040	**Mild**	0.660	0.330	0.010
**Moderate**	0.177	0.607	0.216	**Moderate**	0.190	0.652	0.158
**Severe**	0.009	0.411	0.580	**Severe**	0.011	0.443	0.546
**Milnacipran 100mg (SMD= -0.17)**				**Milnacipran 200mg (SMD=-0.22)**			
From	**To Mild**	**To Moderate**	**To Severe**	From	**To Mild**	**To Moderate**	**To Severe**
**Mild**	0.617	0.309	0.074	**Mild**	0.634	0.317	0.049
**Moderate**	0.167	0.573	0.259	**Moderate**	0.174	0.598	0.228
**Severe**	0.009	0.390	0.601	**Severe**	0.010	0.406	0.584

Abbreviations: SMD, Standardized Mean Difference.

© 2026 Downen SS et al. *JAMA Network Open*.

eTable 2. Direct and Indirect Costs

eTable 2A. Direct Costs

Parameter	Value, mean (SD)	Distribution	Source
**Physician visits**			
Mild	$ 284.45 (309.31)	Gamma	Chandran et al.[2]
Moderate	$ 529.43 (618.62)	Gamma	Chandran et al.[2]
Severe	$ 718.53 (787.34)	Gamma	Chandran et al.[2]
**Diagnostic tests**			
Mild	$ 50.49 (145.03)	Gamma	Chandran et al.[2]
Moderate	$27.96 (137.64)	Gamma	Chandran et al.[2]
Severe	$254.85 (836.17)	Gamma	Chandran et al.[2]
**Emergency visits**			
Mild	$ 0 (0)	Gamma	Chandran et al.[2]
Moderate	$ 21.38 (65.12)	Gamma	Chandran et al.[2]
Severe	$ 19.72 (93.23)	Gamma	Chandran et al.[2]
**Hospitalization**			
Mild	$ 0 (0)	-	Chandran et al.[2]
Moderate	$ 0 (0)	-	Chandran et al.[2]
Severe	$ 0 (0)	-	Chandran et al.[2]
**Out-of-pocket direct costs paid by patient**			
Mild	$ 245.85 (491.34)	Gamma	Chandran et al.[2]
Moderate	$ 495.34 (787.34)	Gamma	Chandran et al.[2]
Severe	$ 613.40 (1290.52)	Gamma	Chandran et al.[2]
**Other medical and nonmedical direct costs**			
Mild	$ 228.81 (606.78)	Gamma	Chandran et al.[2]
Moderate	$ 96.15 (408.47)	Gamma	Chandran et al.[2]
Severe	$ 276.27 (719.25)	Gamma	Chandran et al.[2]

Abbreviations: SD, Standard Deviation.

© 2026 Downen SS et al. *JAMA Network Open*.

eTable 2B. Indirect Costs

Parameter	Value, mean (SD)	Distribution	Source
**Absenteeism**			
Mild	$ 202.25 (587.53)	Gamma	Chandran et al.[2]
Moderate	$ 394.6 (745.89)	Gamma	Chandran et al.[2]
Severe	$ 593.57 (2182.93)	Gamma	Chandran et al.[2]
**Disability payments**			
Mild	$ 0 (0)	Gamma	Chandran et al.[2]
Moderate	$ 403.94 (1232.79)	Gamma	Chandran et al.[2]
Severe	$ 1,192.06 (1912.09)	Gamma	Chandran et al.[2]
**Productivity loss due to disability**			
Mild	$ 0 (0)	Gamma	Chandran et al.[2]
Moderate	$ 1,387.46 (4231.68)	Gamma	Chandran et al.[2]
Severe	$ 4,088.06 (6565.04)	Gamma	Chandran et al.[2]
**Unemployment/ early retirement**			
Mild	$ 1,619.57 (4601.15)	Gamma	Chandran et al.[2]
Moderate	$ 1,734.65 (4193.19)	Gamma	Chandran et al.[2]
Severe	$ 3,535.07 (6168.77)	Gamma	Chandran et al.[2]
**Reduced work schedule**			
Mild	$ 0 (0)	Gamma	Chandran et al.[2]
Moderate	$ 2,107.87 (4129.05)	Gamma	Chandran et al.[2]
Severe	$ 453.81 (1755.21)	Gamma	Chandran et al.[2]
**Unpaid informal care**			
Mild	$ 384.74 (671.90)	Gamma	Chandran et al.[2]
Moderate	$ 2,421.93 (9587.09)	Gamma	Chandran et al.[2]
Severe	$ 3,755.71 (8639.92)	Gamma	Chandran et al.[2]

Abbreviations: SD, Standard Deviation.

© 2026 Downen SS et al. *JAMA Network Open*.

eTable 2C. Drug Daily Costs

Parameter	ɹe, Cost	·ibution	Source
Amitriptyline 25mg	$ 0.29	-	Red Book[3]
Pregabalin 150mg	$ 0.31	-	Red Book[3]
Pregabalin 300mg	$ 0.62	-	Red Book[3]
Pregabalin 450mg	$ 0.92	-	Red Book[3]
Pregabalin 600mg	$ 1.23	-	Red Book[3]
Duloxetine 60mg	$ 0.45	-	Red Book[3]
Duloxetine 120mg	$ 0.89	-	Red Book[3]
Milnacipran 100mg	$ 9.02	-	Red Book[3]
Milnacipran 200mg	$ 18.06	-	Red Book[3]

Abbreviations: SD, Standard Deviation.

© 2026 Downen SS et al. *JAMA Network Open*.

eTable 2D. Total Costs

Parameter	Value, mean (SD)	stribution	Source
**Total 3-months direct costs**			
Mild	$ 976.19 (634.75)	Gamma	Chandran et al.[2]
Moderate	$ 1,377.85 (923.19)	Gamma	Chandran et al.[2]
Severe	$ 2,195.03 (1761.13)	Gamma	Chandran et al.[2]
**Total 3-months indirect costs**			
Mild	$ 2,206.55 (4975.58)	Gamma	Chandran et al.[2]
Moderate	$ 8,450.47 (11656.06)	Gamma	Chandran et al.[2]
Severe	$ 13,618.27 (13529.70)	Gamma	Chandran et al.[2]
**Total annual direct costs**			
Mild	$ 3,904.76 (2539.01)	Gamma	Chandran et al.[2]
Moderate	$ 5,511.38 (3692.75)	Gamma	Chandran et al.[2]
Severe	$ 8,780.10 (7044.53)	Gamma	Chandran et al.[2]
**Total annual indirect costs**			
Mild	$ 8,826.20 (19902.26)	Gamma	Chandran et al.[2]
Moderate	$ 33,801.89 (46623.85)	Gamma	Chandran et al.[2]
Severe	$ 54,473.06 (54118.66)	Gamma	Chandran et al.[2]
**Total 3-months costs**			
Mild	$ 3,182.74 (5610.33)	Gamma	Chandran et al.[2]
Moderate	$ 9,828.32 (12166.63)	Gamma	Chandran et al.[2]
Severe	$ 15,813.3 (15290.83)	Gamma	Chandran et al.[2]
**Total annual costs**			
Mild	$ 12,730.96 (19982.23)	Gamma	Chandran et al.[2]
Moderate	$ 39,313.28 (48666.49)	Gamma	Chandran et al.[2]
Severe	$ 63,253.2 (61162.34)	Gamma	Chandran et al.[2]

Abbreviations: SD, Standard Deviation.

© 2026 Downen SS et al. *JAMA Network Open*.

eTable 3. Utilities

Parameter	Value, mean (SD)	Distribution	Source
Mild	0.76 (0.11)	Lognormal	Schaefer et al.[1]
Moderate	0.57 (0.21)	Lognormal	Schaefer et al.[1]
Severe	0.2 (0.31)	Lognormal	Schaefer et al.[1]

Abbreviations: SD, Standard Deviation.

© 2026 Downen SS et al. *JAMA Network Open*.

eTable 4. 2021 US Life Tables

eTable 4A. US Life Table (General Population)

Age (years)	Probability of dying between ages *x* and *x* + 1	Number surviving to age *x*	Number dying between ages *x* and *x* + 1	Person-years lived between ages *x* and *x* + 1	Total number of person-years lived above age *x*	Expectation of life at age *x*
	q_x	l_x	d_x	L_x	T_x	e_x
0–1	0.005446	100,000	545	99,522	7,637,023	76.4
1–2	0.000403	99,455	40	99,435	7,537,501	75.8
2–3	0.000254	99,415	25	99,403	7,438,065	74.8
3–4	0.000192	99,390	19	99,381	7,338,663	73.8
4–5	0.000161	99,371	16	99,363	7,239,282	72.9
5–6	0.000143	99,355	14	99,348	7,139,919	71.9
6–7	0.000130	99,341	13	99,334	7,040,571	70.9
7–8	0.000119	99,328	12	99,322	6,941,237	69.9
8–9	0.000107	99,316	11	99,311	6,841,915	68.9
9–10	0.000095	99,305	9	99,301	6,742,604	67.9
10–11	0.000090	99,296	9	99,292	6,643,303	66.9
11–12	0.000100	99,287	10	99,282	6,544,012	65.9
12–13	0.000136	99,277	13	99,270	6,444,730	64.9
13–14	0.000205	99,264	20	99,253	6,345,459	63.9
14–15	0.000299	99,243	30	99,228	6,246,206	62.9
15–16	0.000405	99,214	40	99,194	6,146,977	62.0
16–17	0.000513	99,173	51	99,148	6,047,784	61.0
17–18	0.000623	99,123	62	99,092	5,948,636	60.0
18–19	0.000731	99,061	72	99,025	5,849,544	59.1
19–20	0.000837	98,988	83	98,947	5,750,519	58.1
20–21	0.000949	98,906	94	98,859	5,651,572	57.1
21–22	0.001065	98,812	105	98,759	5,552,714	56.2
22–23	0.001170	98,706	115	98,649	5,453,955	55.3
23–24	0.001259	98,591	124	98,529	5,355,306	54.3
24–25	0.001335	98,467	131	98,401	5,256,777	53.4
25–26	0.001406	98,335	138	98,266	5,158,376	52.5
26–27	0.001480	98,197	145	98,125	5,060,110	51.5
27–28	0.001560	98,052	153	97,975	4,961,985	50.6
28–29	0.001651	97,899	162	97,818	4,864,010	49.7
29–30	0.001749	97,737	171	97,652	4,766,191	48.8
30–31	0.001849	97,566	180	97,476	4,668,539	47.8
31–32	0.001947	97,386	190	97,291	4,571,063	46.9
32–33	0.002040	97,196	198	97,097	4,473,772	46.0

© 2026 Downen SS et al. *JAMA Network Open*.

Age (years)	Probability of dying between ages *x* and *x* + 1	Number surviving to age *x*	Number dying between ages *x* and *x* + 1	Person-years lived between ages *x* and *x* + 1	Total number of person-years lived above age *x*	Expectation of life at age *x*
	q_x	l_x	d_x	L_x	T_x	e_x
33–34	0.002128	96,998	206	96,895	4,376,675	45.1
34–35	0.002216	96,792	214	96,685	4,279,780	44.2
35–36	0.002308	96,577	223	96,466	4,183,095	43.3
36–37	0.002409	96,354	232	96,238	4,086,629	42.4
37–38	0.002519	96,122	242	96,001	3,990,391	41.5
38–39	0.002638	95,880	253	95,754	3,894,389	40.6
39–40	0.002768	95,627	265	95,495	3,798,636	39.7
40–41	0.002916	95,363	278	95,223	3,703,141	38.8
41–42	0.003078	95,084	293	94,938	3,607,917	37.9
42–43	0.003244	94,792	307	94,638	3,512,979	37.1
43–44	0.003410	94,484	322	94,323	3,418,342	36.2
44–45	0.003587	94,162	338	93,993	3,324,018	35.3
45–46	0.003792	93,824	356	93,646	3,230,025	34.4
46–47	0.004036	93,468	377	93,280	3,136,379	33.6
47–48	0.004315	93,091	402	92,890	3,043,099	32.7
48–49	0.004625	92,690	429	92,475	2,950,209	31.8
49–50	0.004959	92,261	458	92,032	2,857,733	31.0
50–51	0.005308	91,803	487	91,560	2,765,701	30.1
51–52	0.005686	91,316	519	91,056	2,674,142	29.3
52–53	0.006118	90,797	555	90,519	2,583,085	28.4
53–54	0.006620	90,241	597	89,943	2,492,566	27.6
54–55	0.007184	89,644	644	89,322	2,402,623	26.8
55–56	0.007766	89,000	691	88,654	2,313,301	26.0
56–57	0.008369	88,309	739	87,939	2,224,647	25.2
57–58	0.009042	87,570	792	87,174	2,136,708	24.4
58–59	0.009795	86,778	850	86,353	2,049,534	23.6
59–60	0.010606	85,928	911	85,472	1,963,181	22.8
60–61	0.011467	85,017	975	84,529	1,877,709	22.1
61–62	0.012333	84,042	1,037	83,523	1,793,180	21.3
62–63	0.013173	83,005	1,093	82,458	1,709,656	20.6
63–64	0.013981	81,912	1,145	81,339	1,627,198	19.9
64–65	0.014798	80,767	1,195	80,169	1,545,859	19.1
65–66	0.015666	79,571	1,247	78,948	1,465,690	18.4
66–67	0.016726	78,325	1,310	77,670	1,386,742	17.7
67–68	0.017853	77,015	1,375	76,327	1,309,072	17.0
68–69	0.019122	75,640	1,446	74,917	1,232,745	16.3

© 2026 Downen SS et al. *JAMA Network Open*.

Age (years)	Probability of dying between ages *x* and *x* + 1	Number surviving to age *x*	Number dying between ages *x* and *x* + 1	Person-years lived between ages *x* and *x* + 1	Total number of person-years lived above age *x*	Expectation of life at age *x*
	q_x	l_x	d_x	L_x	T_x	e_x
69–70	0.020526	74,193	1,523	73,432	1,157,828	15.6
70–71	0.021919	72,671	1,593	71,874	1,084,396	14.9
71–72	0.023536	71,078	1,673	70,241	1,012,522	14.2
72–73	0.025372	69,405	1,761	68,524	942,281	13.6
73–74	0.027616	67,644	1,868	66,710	873,756	12.9
74–75	0.029889	65,776	1,966	64,793	807,047	12.3
75–76	0.033726	63,810	2,152	62,734	742,254	11.6
76–77	0.036933	61,658	2,277	60,519	679,520	11.0
77–78	0.041016	59,380	2,436	58,163	619,001	10.4
78–79	0.044758	56,945	2,549	55,671	560,838	9.8
79–80	0.049530	54,396	2,694	53,049	505,168	9.3
80–81	0.054120	51,702	2,798	50,303	452,119	8.7
81–82	0.059483	48,904	2,909	47,449	401,816	8.2
82–83	0.065401	45,995	3,008	44,491	354,367	7.7
83–84	0.072224	42,987	3,105	41,434	309,876	7.2
84–85	0.080609	39,882	3,215	38,275	268,441	6.7
85–86	0.089139	36,667	3,268	35,033	230,167	6.3
86–87	0.099586	33,399	3,326	31,736	195,134	5.8
87–88	0.111021	30,073	3,339	28,403	163,398	5.4
88–89	0.123484	26,734	3,301	25,083	134,995	5.0
89–90	0.137001	23,433	3,210	21,828	109,911	4.7
90–91	0.151584	20,222	3,065	18,690	88,084	4.4
91–92	0.167229	17,157	2,869	15,722	69,394	4.0
92–93	0.183913	14,288	2,628	12,974	53,672	3.8
93–94	0.201590	11,660	2,351	10,485	40,697	3.5
94–95	0.220190	9,310	2,050	8,285	30,213	3.2
95–96	0.239623	7,260	1,740	6,390	21,928	3.0
96–97	0.259772	5,520	1,434	4,803	15,538	2.8
97–98	0.280504	4,086	1,146	3,513	10,735	2.6
98–99	0.301662	2,940	887	2,497	7,222	2.5
99–100	0.323082	2,053	663	1,721	4,725	2.3
100 and olde	1.000000	1,390	1,390	3,004	3,004	2.2

SOURCE: NCHS, National Vital Statistics System, Mortality.[4]

© 2026 Downen SS et al. *JAMA Network Open*.

eTable 4B. US Life Table for Males

Age (years)	Probability of dying between ages *x* and *x* + 1	Number surviving to age *x*	Number dying between ages *x* and *x* + 1	Person-years lived between ages *x* and *x* + 1	Total number of person-years lived above age *x*	Expectation of life at age *x*
	q_x	l_x	d_x	L_x	T_x	e_x
0-1	0.005833	100,000	583	99,489	7,7354,986	73.53
1-2	0.000416	99,417	41	99,396	7,7255,497	73.0
2 -3	0.000274	99,375	27	99,362	7,156,101	72.0
3-4	0.000224	99,348	22	99,337	7,056,739	71.0
4-5	0.000175	99,326	17	99,317	6,957,402	70.0
5-6	0.000161	99,308	16	99,300	6,858,085	69.1
6-7	0.000149	99,292	15	99,285	6,758,785	68.1
7-8	0.000137	99,278	14	99,271	6,659,500	67.1
8-9	0.000119	99,264	12	99,258	6,560,229	66.1
9-10	0.000098	99,252	10	99,247	6,460,971	65.1
10-11	0.000084	99,242	8	99,238	6,361,724	64.1
11-12	0.000093	99,234	9	99,230	6,262,485	63.1
12-13	0.000144	99,225	14	99,218	6,163,256	62.1
13-14	0.000248	99,211	25	99,198	6,064,038	61.1
14-15	0.000392	99,186	39	99,167	5,964,840	60.1
15-16	0.000556	99,147	55	99,120	5,865,673	59.2
16-17	0.000719	99,092	71	99,056	5,766,553	58.2
17-18	0.000885	99,021	88	98,977	5,667,497	57.2
18-19	0.001044	98,933	103	98,882	5,568,520	56.3
19-20	0.001199	98,830	118	98,771	5,469,638	55.3
20-21	0.001361	98,711	134	98,644	5,370,868	54.4
21-22	0.001527	98,577	151	98,502	5,272,223	53.5
22-23	0.001678	98,427	165	98,344	5,173,722	52.6
23-24	0.001805	98,261	177	98,173	5,075,378	51.7
24-25	0.001915	98,084	188	97,990	4,977,205	50.7
25-26	0.002015	97,896	197	97,798	4,879,215	49.8
26-27	0.002116	97,699	207	97,596	4,781,417	48.9
27-28	0.002221	97,492	217	97,384	4,683,821	48.0
28-29	0.002334	97,276	227	97,162	4,586,438	47.1
29-30	0.002451	97,049	238	96,930	4,489,275	46.3
30-31	0.002569	96,811	249	96,686	4,392,346	45.4
31-32	0.002682	96,562	259	96,433	4,295,659	44.5
32-33	0.002789	96,303	269	96,169	4,199,227	43.6
Age (years)	Probability of dying between	Number surviving to age *x*	Number dying between	Person-years lived	Total number of person-	Expectation of life at age *x*

© 2026 Downen SS et al. *JAMA Network Open*.

	ages *x* and *x* + 1		ages *x* and *x* + 1	between ages *x* and *x* + 1	years lived above age *x*	
	q_x	l_x	d_x	L_x	T_x	e_x
33-34	0.002887	96,035	277	95,896	4,103,058	42.7
34-35	0.002982	95,757	286	95,615	4,007,162	41.8
35-36	0.003081	95,472	294	95,325	3,911,547	41.0
36-37	0.003190	95,178	304	95,026	3,816,222	40.1
37-38	0.003310	94,874	314	94,717	3,721,196	39.2
38-39	0.003446	94,560	326	94,397	3,626,479	38.4
39-40	0.003597	94,234	339	94,065	3,532,082	37.5
40-41	0.003772	93,895	354	93,718	3,438,018	36.6
41-42	0.003964	93,541	371	93,356	3,344,299	35.8
42-43	0.004158	93,170	387	92,977	3,250,944	34.9
43-44	0.004353	92,783	404	92,581	3,157,967	34.0
44-45	0.004560	92,379	421	92,168	3,065,386	33.2
45-46	0.004799	91,958	441	91,737	2,973,218	32.3
46-47	0.005090	91,516	466	91,283	2,881,481	31.5
47-48	0.005431	91,051	494	90,803	2,790,198	30.6
48-49	0.005818	90,556	527	90,293	2,699,394	29.8
49-50	0.006241	90,029	562	89,748	2,609,102	29.0
50-51	0.006679	89,467	598	89,169	2,519,353	28.2
51-52	0.007151	88,870	636	88,552	2,430,185	27.3
52-53	0.007690	88,234	678	87,895	2,341,633	26.5
53-54	0.008316	87,556	728	87,192	2,253,738	25.7
54-55	0.009023	86,828	783	86,436	2,166,546	25.0
55-56	0.009754	86,044	839	85,624	2,080,111	24.2
56-57	0.010510	85,205	895	84,757	1,994,486	23.4
57-58	0.011350	84,309	957	83,831	1,909,729	22.7
58-59	0.012285	83,352	1,024	82,840	1,825,898	21.9
59-60	0.013286	82,328	1,094	81,782	1,743,058	21.2
60-61	0.014341	81,235	1,165	80,652	1,661,276	20.5
61-62	0.015402	80,070	1,233	79,453	1,580,624	19.7
62-63	0.016437	78,836	1,296	78,189	1,501,171	19.0
63-64	0.017445	77,541	1,353	76,864	1,422,982	18.4
64-65	0.018475	76,188	1,408	75,484	1,346,118	17.7
65-66	0.019576	74,780	1,464	74,048	1,270,634	17.0
66-67	0.020927	73,316	1,534	72,549	1,196,586	16.3
67-68	0.022303	71,782	1,601	70,982	1,124,036	15.7

© 2026 Downen SS et al. *JAMA Network Open*.

Age (years)	Probability of dying between ages *x* and *x* + 1	Number surviving to age *x*	Number dying between ages *x* and *x* + 1	Person-years lived between ages *x* and *x* + 1	Total number of person-years lived above age *x*	Expectation of life at age *x*
	q_x	l_x	d_x	L_x	T_x	e_x
68-69	0.023804	70,181	1,671	69,346	1,053,055	15.0
69-70	0.025383	68,511	1,739	67,641	983,709	14.4
70-71	0.026908	66,772	1,797	65,873	916,068	13.7
71-72	0.028704	64,975	1,865	64,042	850,195	13.1
72-73	0.030788	63,110	1,943	62,138	786,152	12.5
73-74	0.033361	61,167	2,041	60,147	724,014	11.8
74-75	0.035944	59,126	2,125	58,064	663,867	11.2
75-76	0.040497	57,001	2,308	55,847	605,804	10.6
76-77	0.044053	54,693	2,409	53,488	549,957	10.1
77-78	0.048810	52,283	2,552	51,007	496,469	9.5
78-79	0.053173	49,731	2,644	48,409	445,461	9.0
79-80	0.058908	47,087	2,774	45,700	397,052	8.4
80-81	0.063954	44,313	2,834	42,896	351,352	7.9
81-82	0.070311	41,479	2,916	40,021	308,456	7.4
82-83	0.076958	38,563	2,968	37,079	268,435	7.0
83-84	0.084813	35,595	3,019	34,086	231,356	6.5
84-85	0.094500	32,576	3,078	31,037	197,271	6.1
85-86	0.104319	29,498	3,077	27,959	166,234	5.6
86-87	0.116428	26,421	3,076	24,882	138,275	5.2
87-88	0.129619	23,344	3,026	21,831	113,392	4.9
88-89	0.143914	20,319	2,924	18,856	91,561	4.5
89-90	0.159317	17,394	2,771	16,009	72,704	4.2
90-91	0.175814	14,623	2,571	13,338	56,695	3.9
91-92	0.193369	12,052	2,331	10,887	43,358	3.6
92-93	0.211919	9,722	2,060	8,692	32,471	3.3
93-94	0.231379	7,661	1,773	6,775	23,779	3.1
94-95	0.251638	5,889	1,482	5,148	17,004	2.9
95-96	0.272559	4,407	1,201	3,806	11,856	2.7
96-97	0.293988	3,206	942	2,735	8,050	2.5
97-98	0.315751	2,263	715	1,906	5,315	2.3
98-99	0.337666	1,549	523	1,287	3,409	2.2
99-100	0.359544	1,026	369	841	2,122	2.1
100 and older	1.000000	657	657	1,281	1,281	1.9

Source: NCHS, National Vital Statistics System, Mortality.[4]

© 2026 Downen SS et al. *JAMA Network Open*.

© 2026 Downen SS et al. *JAMA Network Open*.

eTable 4C. US Life Table for Females

Age (years)	Probability of dying between ages *x* and *x* + 1	Number surviving to age *x*	Number dying between ages *x* and *x* + 1	Person-years lived between ages *x* and *x* + 1	Total number of person-years lived above age *x*	Expectation of life at age *x*
	q_x	l_x	d_x	L_x	T_x	e_x
0-1	0.005040	100,000	504	99,557	7,932,807	79.3
1-2	0.000389	99,496	39	99,477	7,833,250	78.7
2 -3	0.000234	99,457	23	99,446	7,733,774	77.8
3-4	0.000158	99,434	16	99,426	7,634,328	76.8
4-5	0.000147	99,418	15	99,411	7,534,902	75.8
5-6	0.000124	99,404	12	99,398	7,435,491	74.8
6-7	0.000109	99,391	11	99,386	7,336,093	73.8
7-8	0.000100	99,381	10	99,376	7,236,707	72.8
8-9	0.000094	99,371	9	99,366	7,137,332	71.8
9-10	0.000093	99,361	9	99,357	7,037,966	70.8
10-11	0.000096	99,352	10	99,347	6,938,609	69.8
11-12	0.000107	99,342	11	99,337	6,839,262	68.8
12-13	0.000128	99,332	13	99,325	6,739,925	67.9
13-14	0.000160	99,319	16	99,311	6,640,600	66.9
14-15	0.000201	99,303	20	99,293	6,541,289	65.9
15-16	0.000247	99,283	25	99,271	6,441,995	64.9
16-17	0.000297	99,259	29	99,244	6,342,724	63.9
17-18	0.000349	99,229	35	99,212	6,243,481	62.9
18-19	0.000403	99,195	40	99,175	6,144,269	61.9
19-20	0.000459	99,155	46	99,132	6,045,094	61.0
20-21	0.000520	99,109	52	99,083	5,945,962	60.0
21-22	0.000584	99,057	58	99,029	5,846,879	59.0
22-23	0.000641	99,000	64	98,968	5,747,851	58.1
23-24	0.000690	98,936	68	98,902	5,648,883	57.1
24-25	0.000733	98,868	72	98,832	5,549,981	56.1
25-26	0.000774	98,795	76	98,757	5,451,149	55.2
26-27	0.000820	98,719	81	98,678	5,352,392	54.2
27-28	0.000876	98,638	86	98,595	5,253,714	53.3
28-29	0.000946	98,552	93	98,505	5,155,119	52.3
29-30	0.001026	98,458	101	98,408	5,056,614	51.4
30-31	0.001111	98,357	109	98,303	4,958,206	50.4
31-32	0.001194	98,248	117	98,189	4,859,903	49.5
32-33	0.001275	98,131	125	98,068	4,761,714	48.5
33-34	0.001354	98,006	133	97,939	4,663,646	47.6

Age (years)	Probability of dying between ages *x* and *x* + 1	Number surviving to age *x*	Number dying between ages *x* and *x* + 1	Person-years lived between ages *x* and *x* + 1	Total number of person-years lived above age *x*	Expectation of life at age *x*
	q_x	l_x	d_x	L_x	T_x	e_x
34-35	0.001433	97,873	140	97,803	4,565,707	46.6
35-36	0.001518	97,733	148	97,658	4,467,904	45.7
36-37	0.001611	97,584	157	97,506	4,370,245	44.8
37-38	0.001709	97,427	167	97,344	4,272,740	43.9
38-39	0.001814	97,261	176	97,172	4,175,396	42.9
39-40	0.001925	97,084	187	96,991	4,078,223	42.0
40-41	0.002048	96,897	198	96,798	3,981,232	41.1
41-42	0.002183	96,699	211	96,593	3,884,434	40.2
42-43	0.002321	96,488	224	96,376	3,787,841	39.3
43-44	0.002461	96,264	237	96,145	3,691,465	38.3
44-45	0.002611	96,027	251	95,902	3,595,320	37.4
45-46	0.002784	95,776	267	95,643	3,499,418	36.5
46-47	0.002984	95,510	285	95,367	3,403,775	35.6
47-48	0.003203	95,225	305	95,072	3,308,408	34.7
48-49	0.003436	94,919	326	94,756	3,213,336	33.9
49-50	0.003680	94,593	348	94,419	3,118,580	33.0
50-51	0.003935	94,245	371	94,060	3,024,160	32.1
51-52	0.004217	93,874	396	93,677	2,930,101	31.2
52-53	0.004544	93,479	425	93,266	2,836,424	30.3
53-54	0.004928	93,054	459	92,824	2,743,158	29.5
54-55	0.005361	92,595	496	92,347	2,650,333	28.6
55-56	0.005809	92,099	535	91,831	2,557,986	27.8
56-57	0.006274	91,564	574	91,277	2,466,155	26.9
57-58	0.006795	90,989	618	90,680	2,374,879	26.1
58-59	0.007383	90,371	667	90,037	2,284,199	25.3
59-60	0.008020	89,704	719	89,344	2,194,161	24.5
60-61	0.008703	88,984	774	88,597	2,104,817	23.7
61-62	0.009396	88,210	829	87,796	2,016,220	22.9
62-63	0.010066	87,381	880	86,941	1,928,424	22.1
63-64	0.010706	86,502	926	86,039	1,841,482	21.3
64-65	0.011354	85,576	972	85,090	1,755,444	20.5
65-66	0.012041	84,604	1,019	84,095	1,670,354	19.7
66-67	0.012880	83,585	1,077	83,047	1,586,259	19.0
67-68	0.013821	82,509	1,140	81,938	1,503,212	18.2
68-69	0.014915	81,368	1,214	80,762	1,421,274	17.5

© 2026 Downen SS et al. *JAMA Network Open*.

Age (years)	Probability of dying between ages *x* and *x* + 1	Number surviving to age *x*	Number dying between ages *x* and *x* + 1	Person-years lived between ages *x* and *x* + 1	Total number of person-years lived above age *x*	Expectation of life at age *x*
	q_x	l_x	d_x	L_x	T_x	e_x
69-70	0.016188	80,155	1,298	79,506	1,340,512	16.7
70-71	0.017475	78,857	1,378	78,168	1,261,007	16.0
71-72	0.018964	77,479	1,469	76,744	1,182,838	15.3
72-73	0.020616	76,010	1,567	75,226	1,106,094	14.6
73-74	0.022603	74,443	1,683	73,602	1,030,868	13.8
74-75	0.024647	72,760	1,793	71,864	957,266	13.2
75-76	0.027933	70,967	1,982	69,976	885,402	12.5
76-77	0.030922	68,985	2,133	67,918	815,427	11.8
77-78	0.034536	66,851	2,309	65,697	747,509	11.2
78-79	0.037857	64,543	2,443	63,321	681,812	10.6
79-80	0.041967	62,099	2,606	60,796	618,491	10.0
80-81	0.046336	59,493	2,757	58,115	557,695	9.4
81-82	0.051084	56,736	2,898	55,287	499,580	8.8
82-83	0.056608	53,838	3,048	52,314	444,293	8.3
83-84	0.062881	50,790	3,194	49,194	391,978	7.7
84-85	0.070582	47,597	3,359	45,917	342,785	7.2
85-86	0.079149	44,237	3,501	42,487	296,868	6.7
86-87	0.087870	40,736	3,579	38,946	254,381	6.2
87-88	0.098712	37,156	3,668	35,323	215,435	5.8
88-89	0.110635	33,489	3,705	31,636	180,113	5.4
89-90	0.123686	29,784	3,684	27,942	148,477	5.0
90-91	0.137893	26,100	3,599	24,300	120,535	4.6
91-92	0.153272	22,501	3,449	20,776	96,235	4.3
92-93	0.169816	19,052	3,235	17,434	75,458	4.0
93-94	0.187493	15,817	2,966	14,334	58,024	3.7
94-95	0.206246	12,851	2,651	11,526	43,690	3.4
95-96	0.225991	10,201	2,305	9,048	32,164	3.2
96-97	0.246613	7,895	1,947	6,922	23,116	2.9
97-98	0.267972	5,948	1,594	5,151	16,194	2.7
98-99	0.289905	4,354	1,262	3,723	11,043	2.5
99-100	0.312227	3,092	965	2,609	7,319	2.4
100 and older	1.000000	2,127	2,127	4,710	4,710	2.2

Source: NCHS, National Vital Statistics System, Mortality.[4]

© 2026 Downen SS et al. *JAMA Network Open*.

eFigure 1. Markov Traces of the Included Treatments for Fibromyalgia

eFigure 1A. Trace Line of an Untreated Fibromyalgia Patient

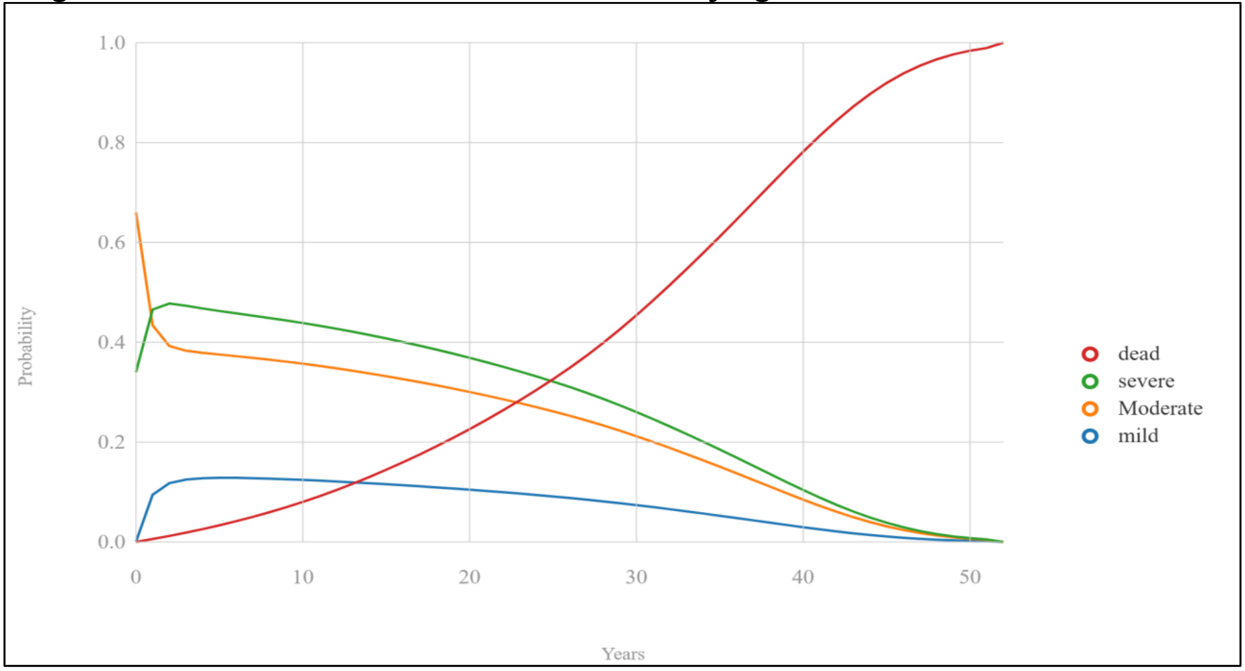

eFigure 1B. Trace Line of the Off-label Use of Amitriptyline

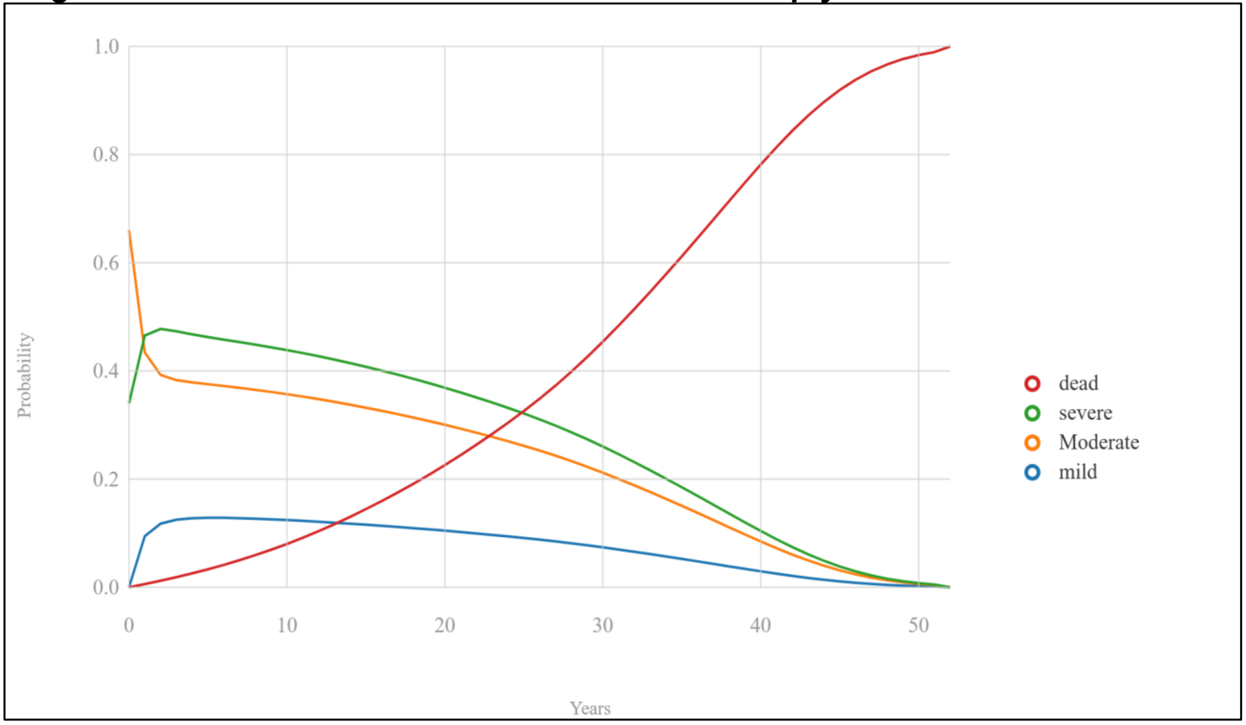

eFigure 1C. Trace Line of Pregabalin 150mg

© 2026 Downen SS et al. *JAMA Network Open*.

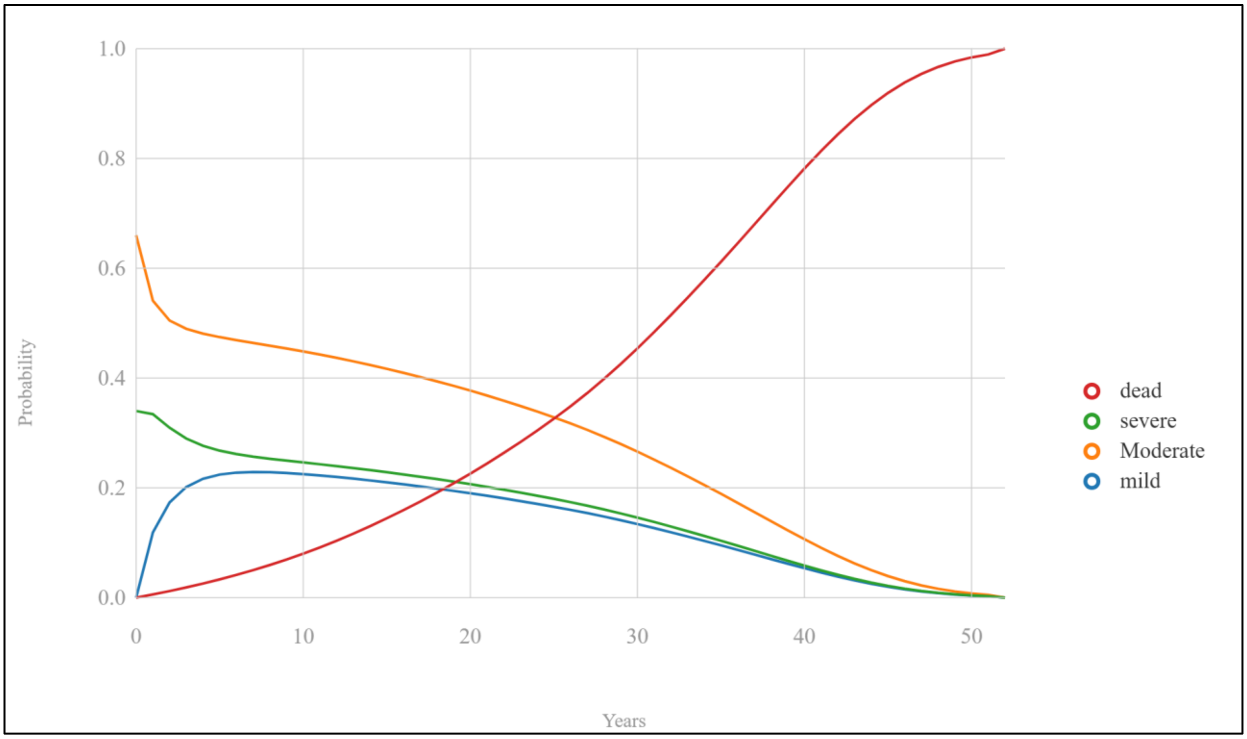

eFigure 1D. Trace Line of Pregabalin 300mg

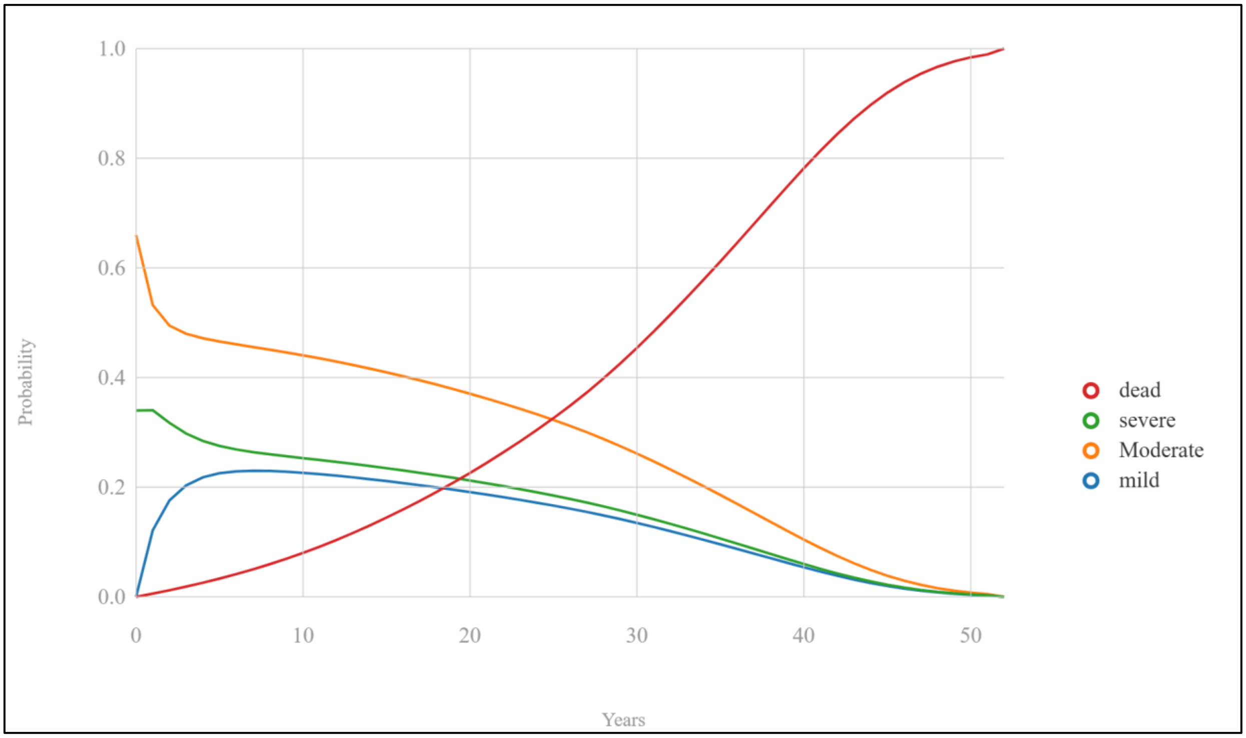

eFigure 1E. Trace Line of Pregabalin 450mg

© 2026 Downen SS et al. *JAMA Network Open*.

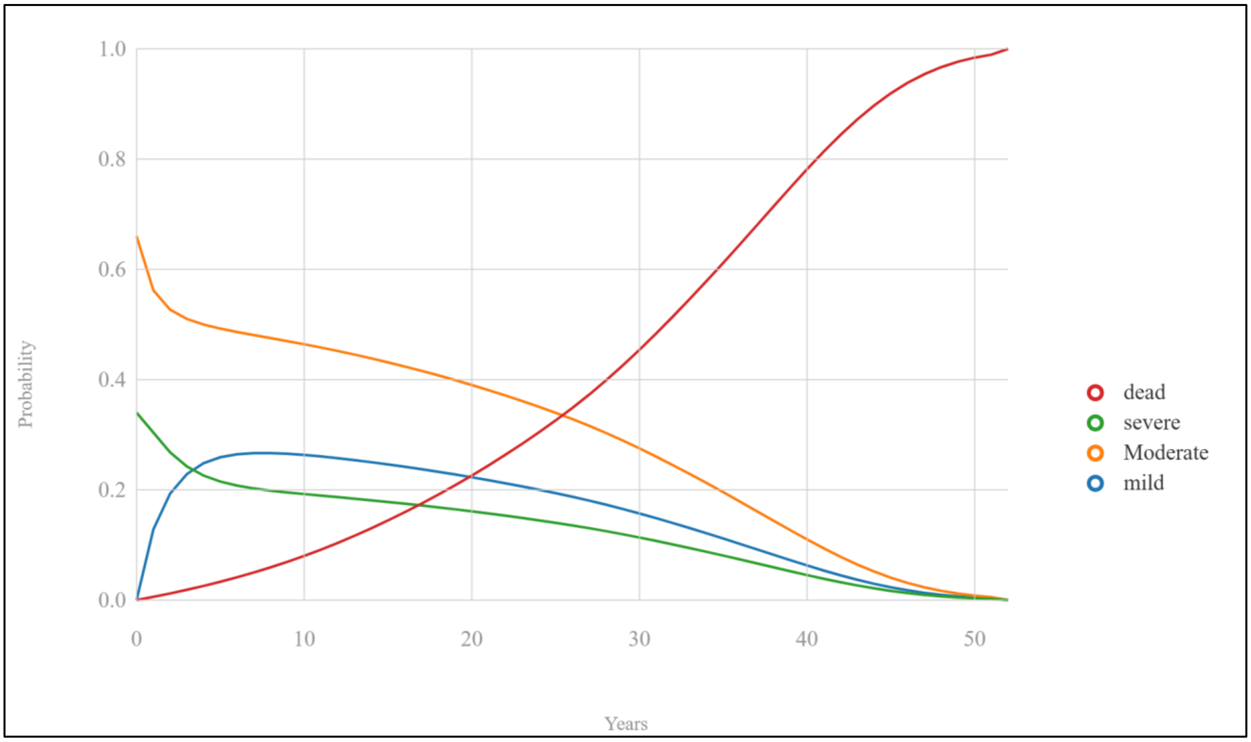

eFigure 1F. Trace Line of Pregabalin 600mg

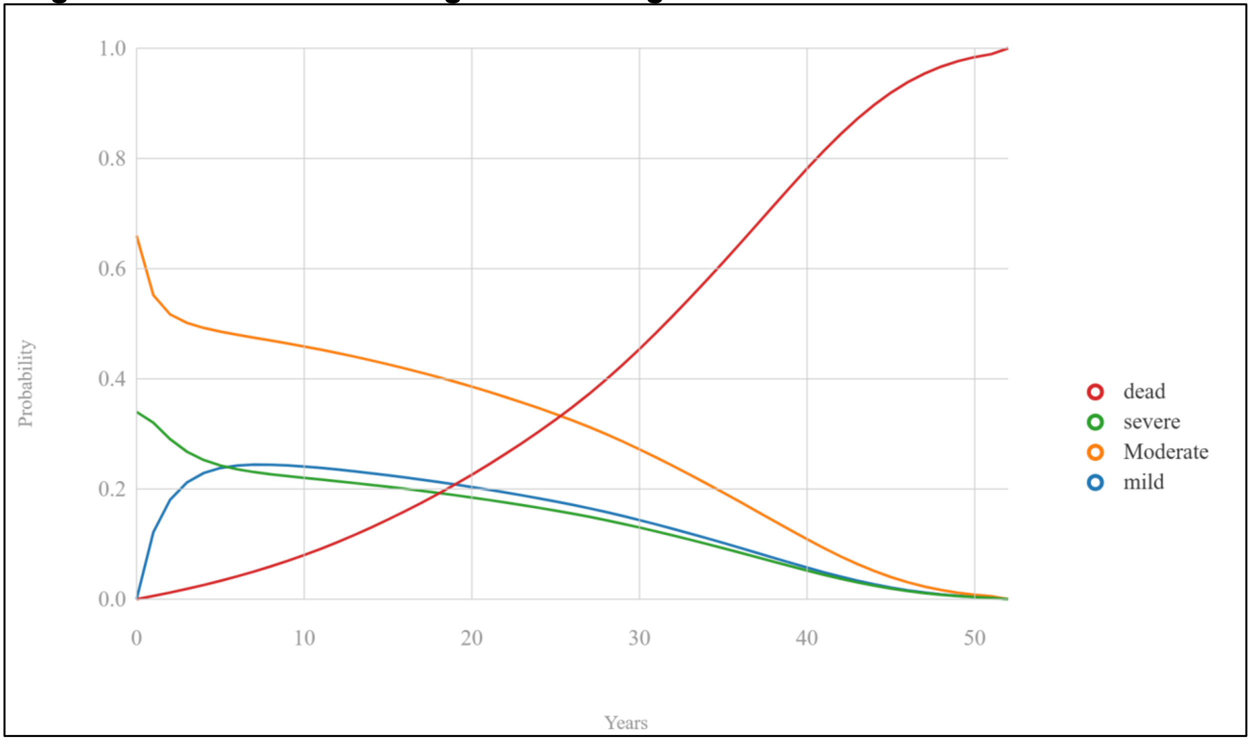

eFigure 1G. Trace Line of Duloxetine 60mg

© 2026 Downen SS et al. *JAMA Network Open*.

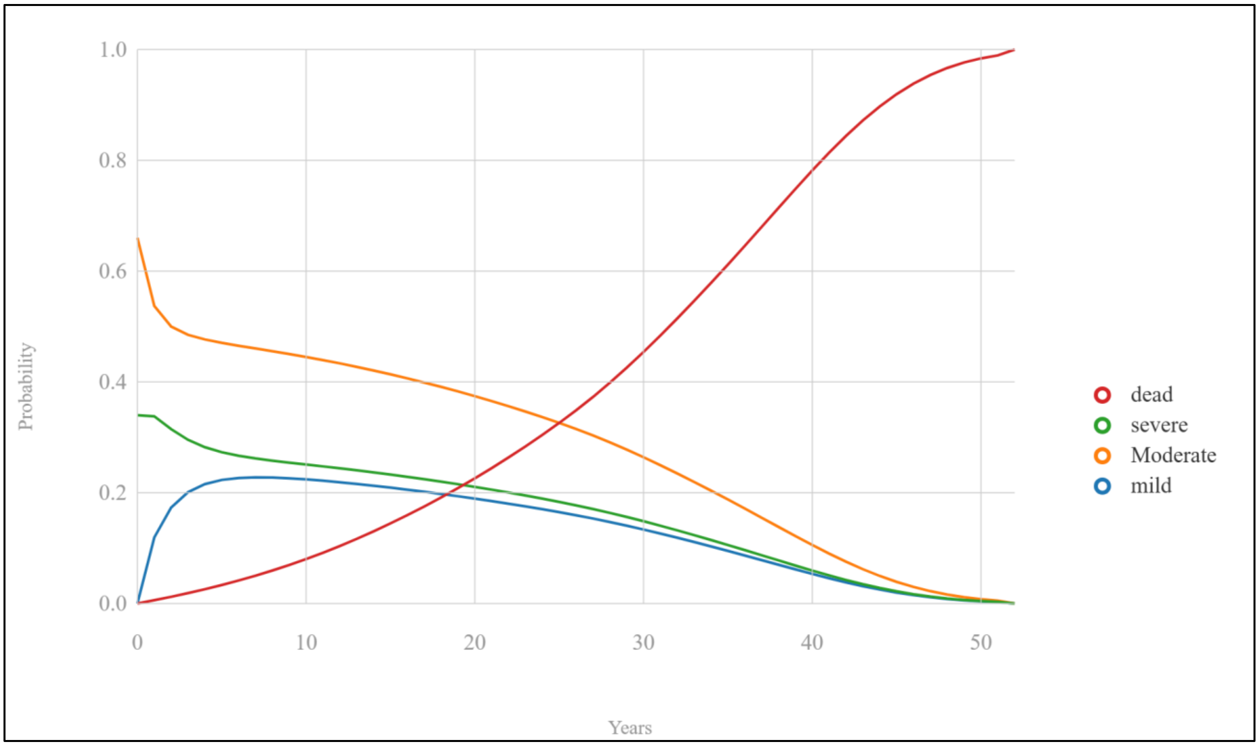

eFigure 1H. Trace Line of Duloxetine 120mg

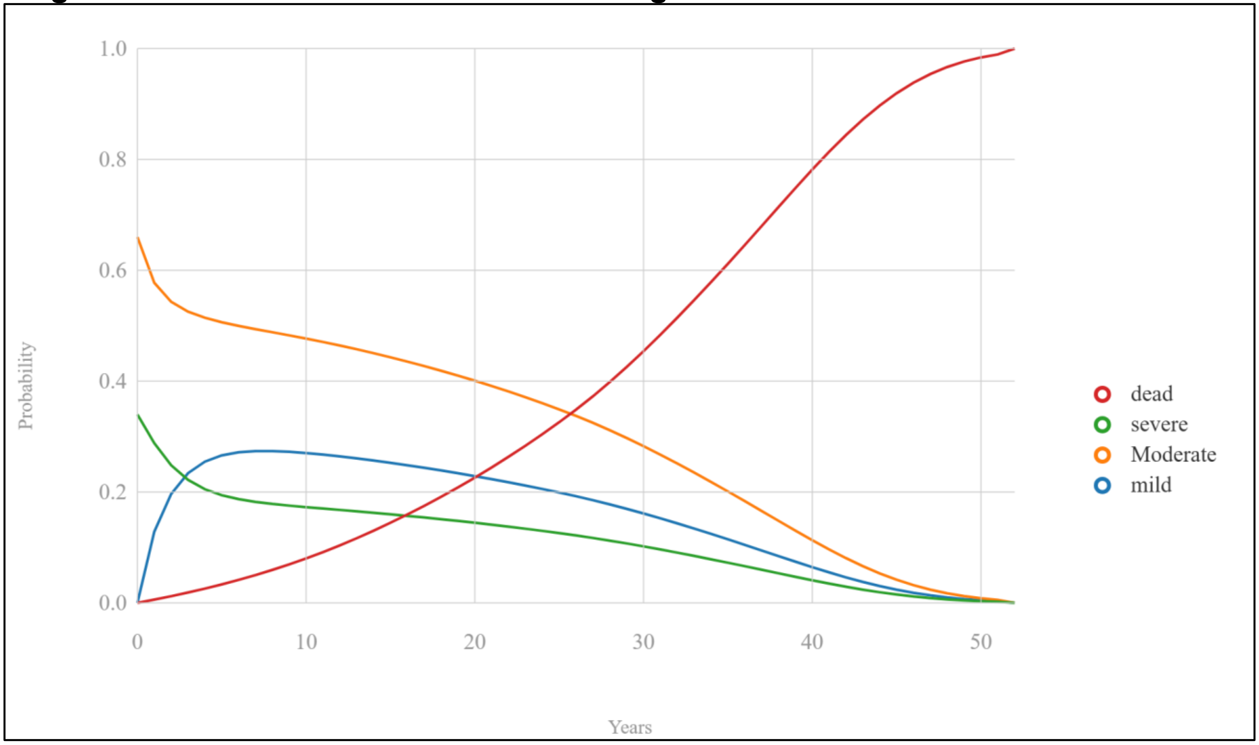

eFigure 1I. Trace Line of Milnacipran 100mg

© 2026 Downen SS et al. *JAMA Network Open*.

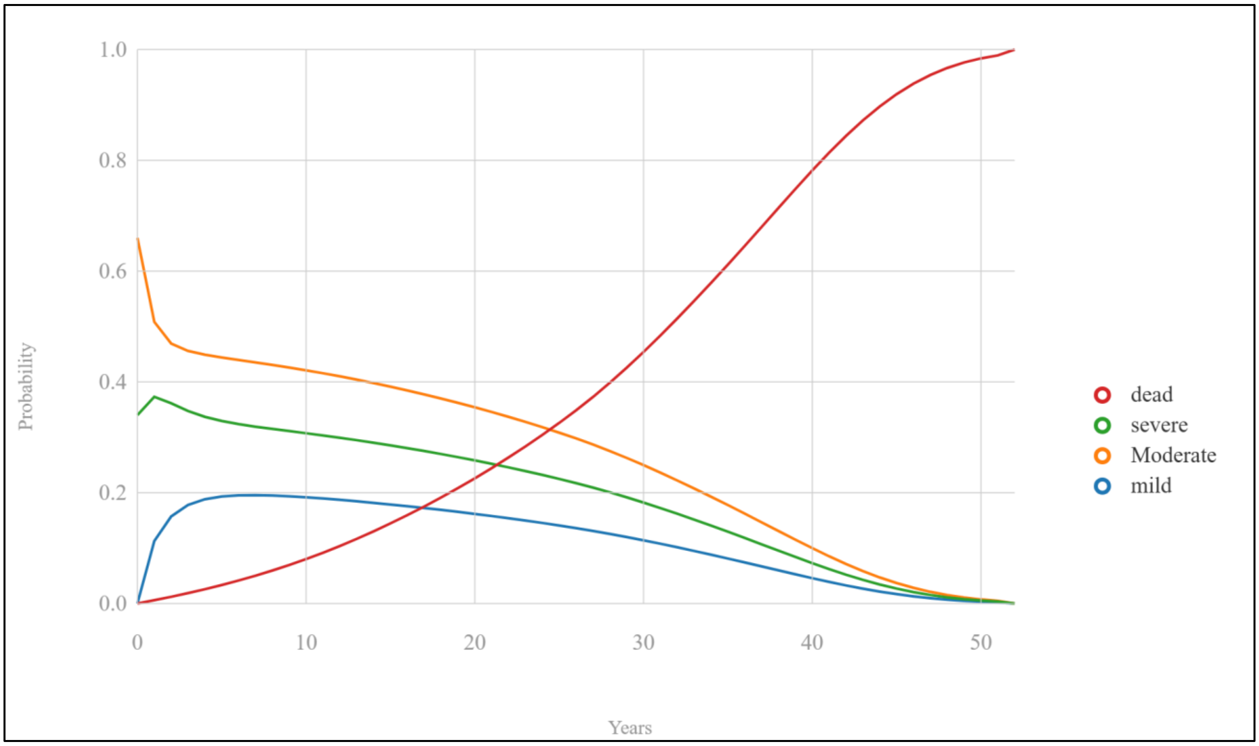

eFigure 1J. Trace Line of Milnacipran 200mg

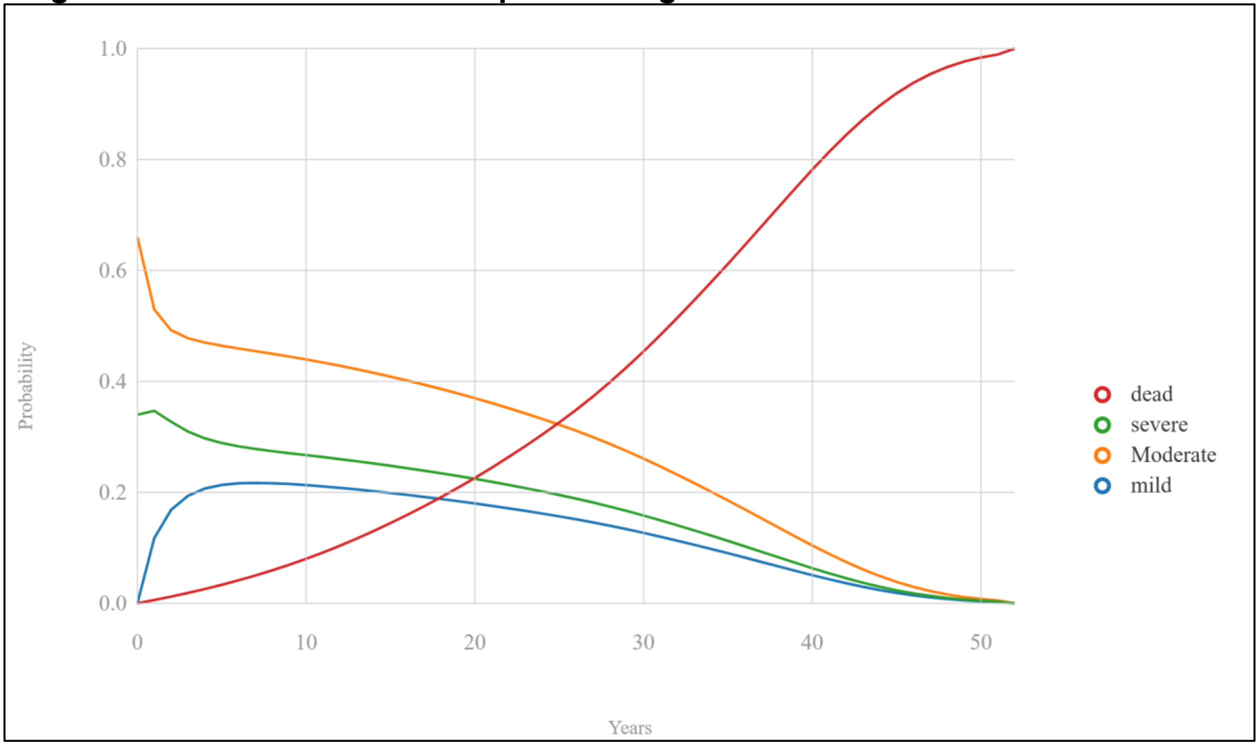

© 2026 Downen SS et al. *JAMA Network Open*.

eFigure 2. Tornado Diagram of Deterministic Sensitivity Analyses, US Health Care Payer Perspective

eFigure 2A. Duloxetine 60mg vs. Amitriptyline

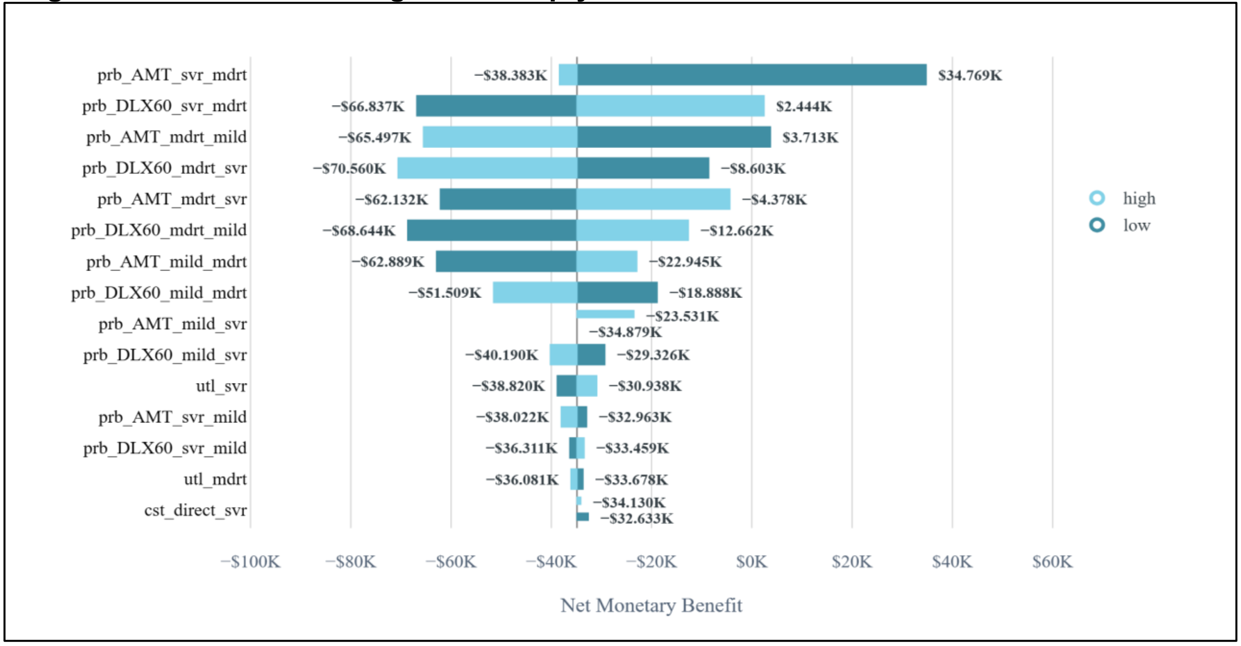

Abbreviations: AMT, Amitriptyline; DLX_60, Duloxetine 60mg; mild_mdrt, transition probability of a fibromyalgia patient from a mild to moderate health state; mild_svr, mild to severe; prb, probabillity; mdrt_mild, moderate to mild; mdrt_svr, moderate to severe; svr_mild, severe to mild; svr_mdrt, severe to moderate; utl_mdrt, utility moderate; utl_svr, utility severe; cst_direct_svr, direct cost (severe state).

eFigure 2B. Duloxetine 120mg vs. Amitriptyline

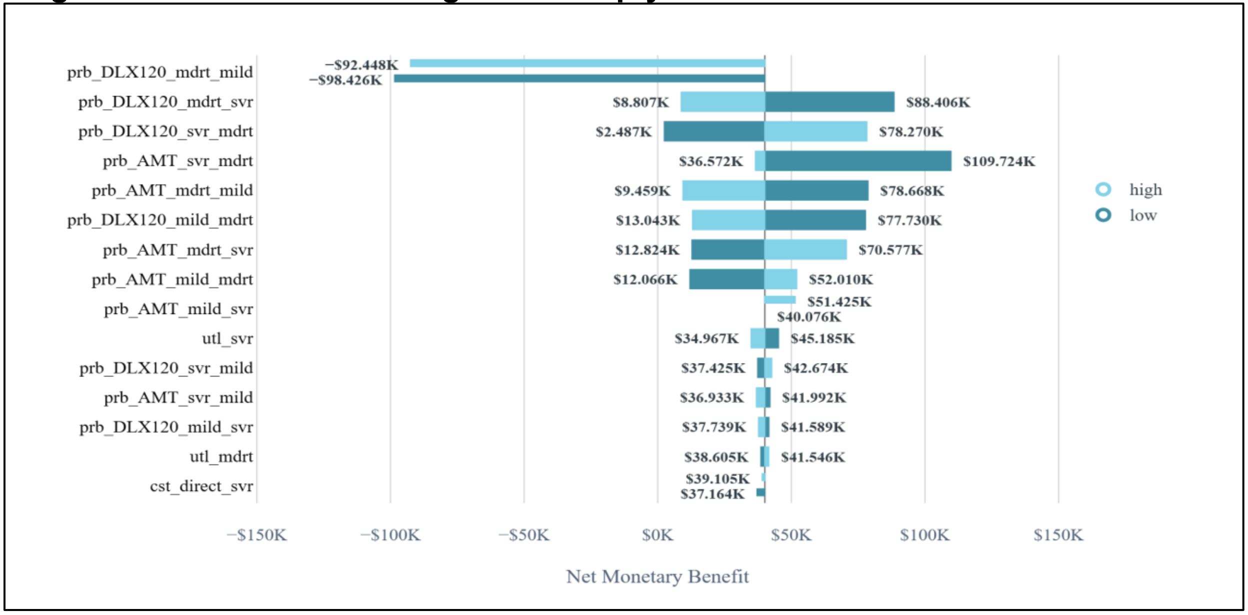

© 2026 Downen SS et al. *JAMA Network Open*.

Abbreviations: AMT, Amitriptyline; DLX_120, Duloxetine 120mg; mild_mdrt, mild to moderate; mild_svr, mild to severe; prb, probabillity; mdrt_mild, moderate to mild; mdrt_svr, moderate to severe; svr_mild, severe to mild; svr_mdrt, severe to moderate; utl_mdrt, utility moderate; utl_svr, utility severe; cst_direct_svr, direct cost (severe state).

eFigure 2C. Milnacipran 100mg vs. Amitriptyline

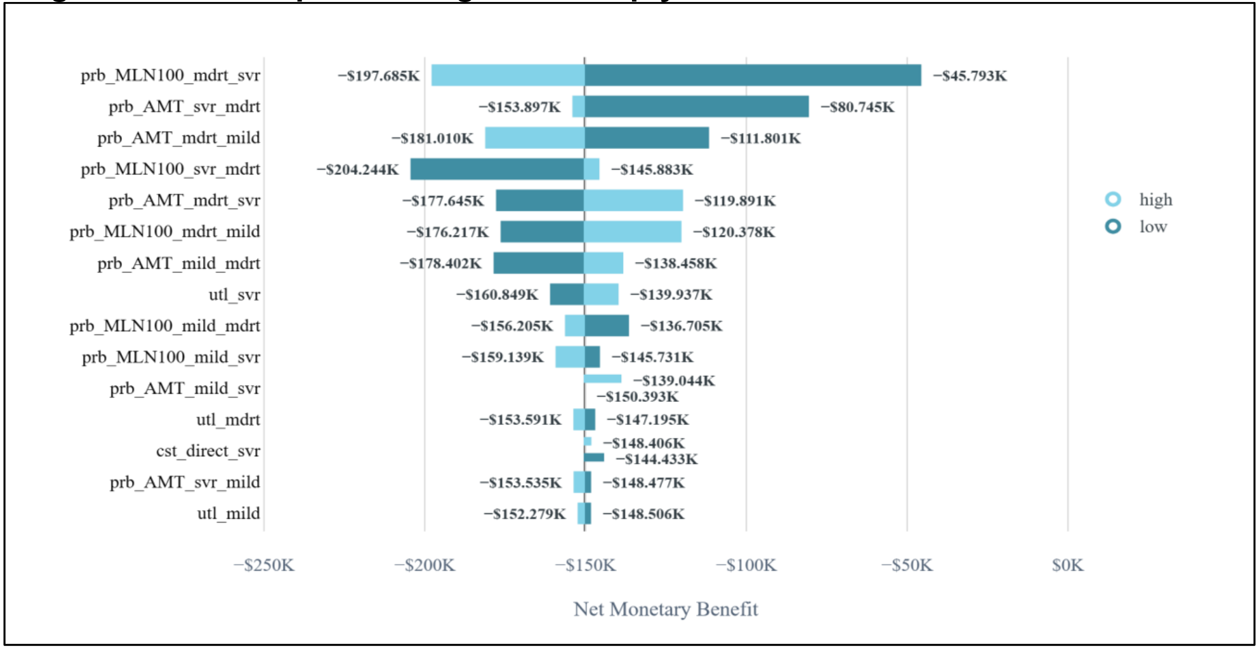

Abbreviations: AMT, Amitriptyline; MLN_100, Milnacipran 100mg; mild_mdrt, mild to moderate; mild_svr, mild to severe; prb, probabillity; mdrt_mild, moderate to mild; mdrt_svr, moderate to severe; svr_mild, severe to mild; svr_mdrt, severe to moderate; utl_mdrt, utility moderate; utl_svr, utility severe; cst_direct_svr, direct cost (severe state).

eFigure 2D. Milnacipran 200mg vs. Amitriptyline

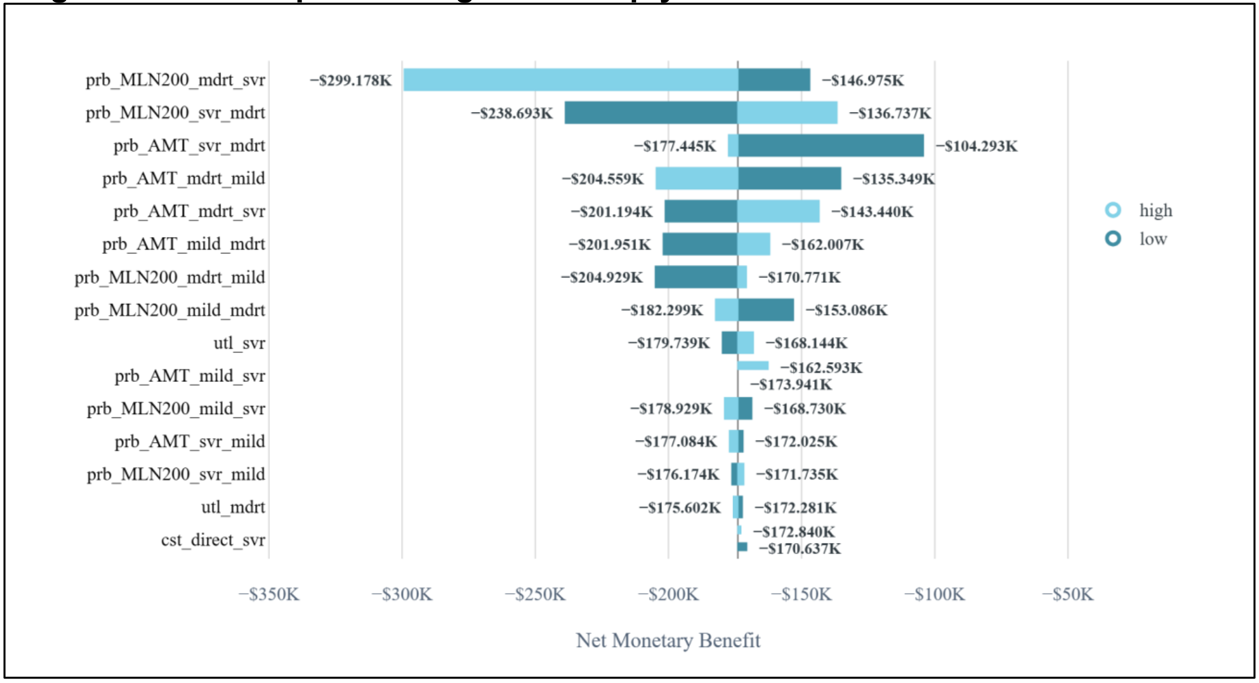

Abbreviations: AMT, Amitriptyline; MLN200, Milnacipran 200mg; mild_mdrt, mild to moderate; mild_svr, mild to severe; prb, probabillity; mdrt_mild, moderate to mild; mdrt_svr, moderate to severe; svr_mild, severe to mild; svr_mdrt, severe to moderate; utl_mdrt, utility moderate; utl_svr, utility severe; cst_direct_svr, direct cost (severe state).

© 2026 Downen SS et al. *JAMA Network Open*.

© 2026 Downen SS et al. *JAMA Network Open*.

eFigure 2E. Pregabalin 150mg vs. Amitriptyline

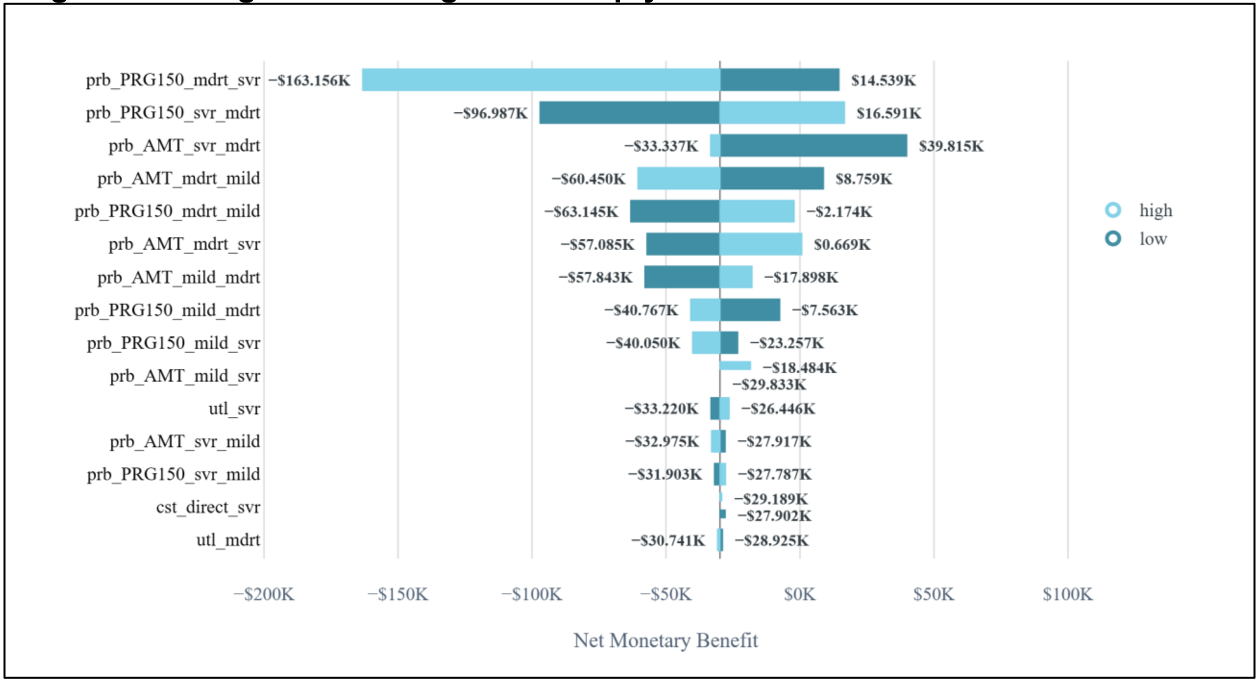

Abbreviations: AMT, Amitriptyline; PRG_150, Pregabalin 150mg; mild_mdrt, mild to moderate; mild_svr, mild to severe; prb, probabillity; mdrt_mild, moderate to mild; mdrt_svr, moderate to severe; svr_mild, severe to mild; svr_mdrt, severe to moderate; utl_mdrt, utility moderate; utl_svr, utility severe; cst_direct_svr, direct cost (severe state).

eFigure 2F. Pregabalin 300mg vs. Amitriptyline

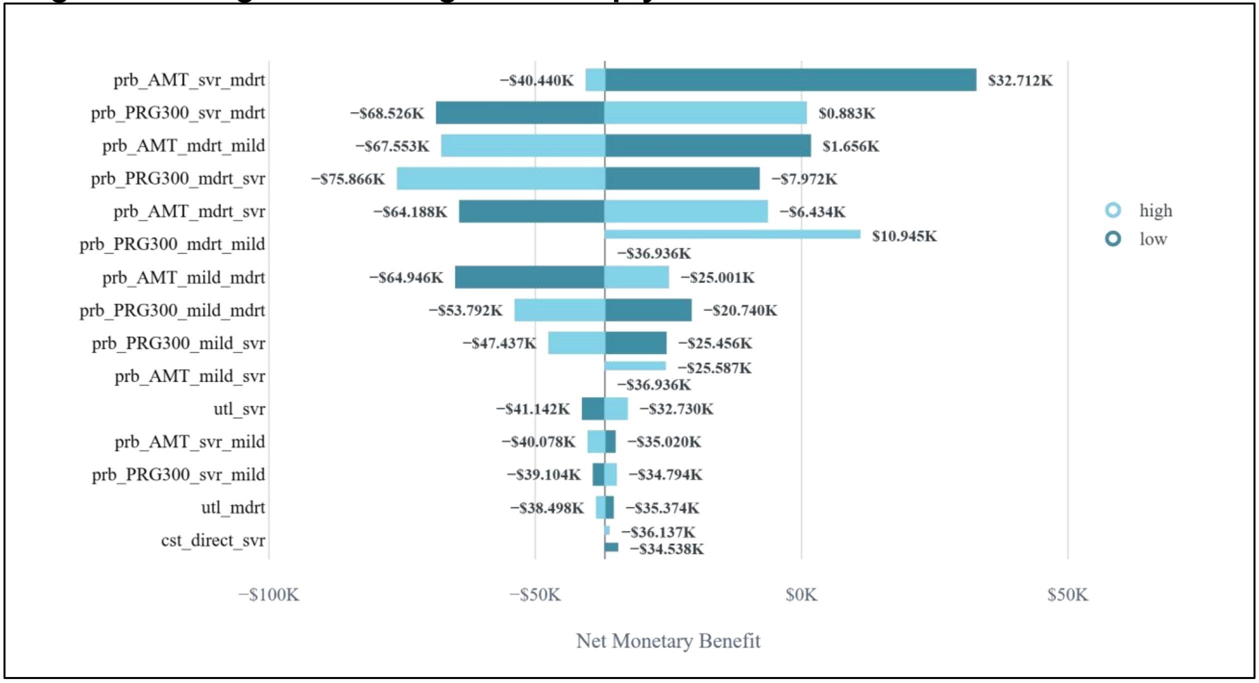

Abbreviations: AMT, Amitriptyline; PRG_300, Pregabalin 300mg; mild_mdrt, mild to moderate; mild_svr, mild to severe; prb, probabillity; mdrt_mild, moderate to mild; mdrt_svr, moderate to severe; svr_mild, severe to mild; svr_mdrt, severe to moderate; utl_mdrt, utility moderate; utl_svr, utility severe; cst_direct_svr, direct cost (severe state).

© 2026 Downen SS et al. *JAMA Network Open*.

eFigure 2G. Pregabalin 450mg vs. Amitriptyline

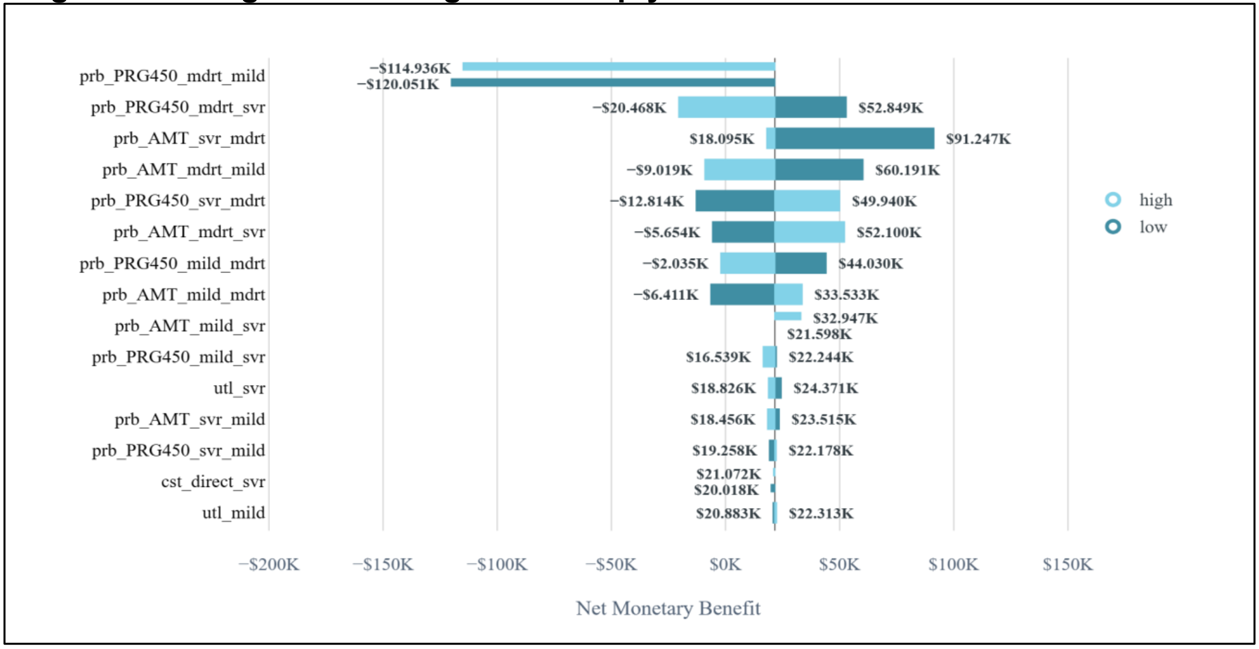

Abbreviations: AMT, Amitriptyline; PRG_450, Pregabalin 450mg; mild_mdrt, mild to moderate; mild_svr, mild to severe; prb, probabillity; mdrt_mild, moderate to mild; mdrt_svr, moderate to severe; svr_mild, severe to mild; svr_mdrt, severe to moderate; utl_mdrt, utility moderate; utl_svr, utility severe; cst_direct_svr, direct cost (severe state).

eFigure 2H. Pregabalin 600mg vs. Amitriptyline

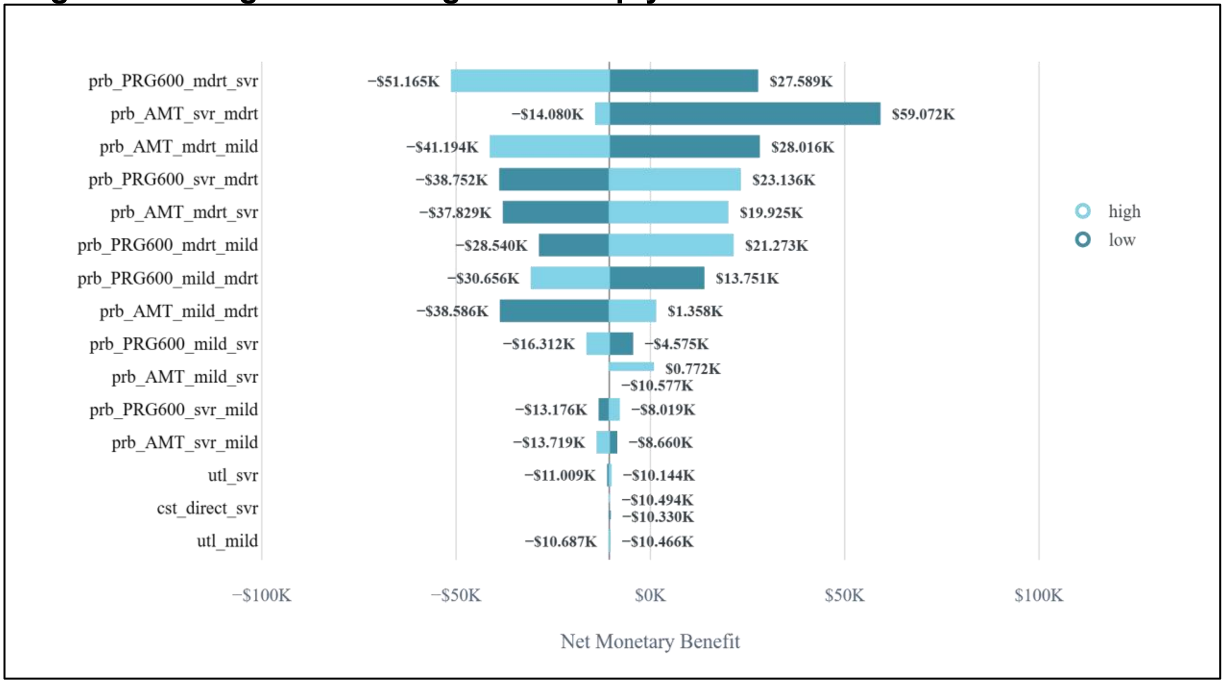

Abbreviations: AMT, Amitriptyline; PRG_600, Pregabalin 600mg; mild_mdrt, mild to moderate; mild_svr, mild to severe; prb, probabillity; mdrt_mild, moderate to mild; mdrt_svr, moderate to severe; svr_mild, severe to mild; svr_mdrt, severe to moderate; utl_mdrt, utility moderate; utl_svr, utility severe; cst_direct_svr, direct cost (severe state).

© 2026 Downen SS et al. *JAMA Network Open*.

eFigure 2I. No treatment vs. Amitriptyline

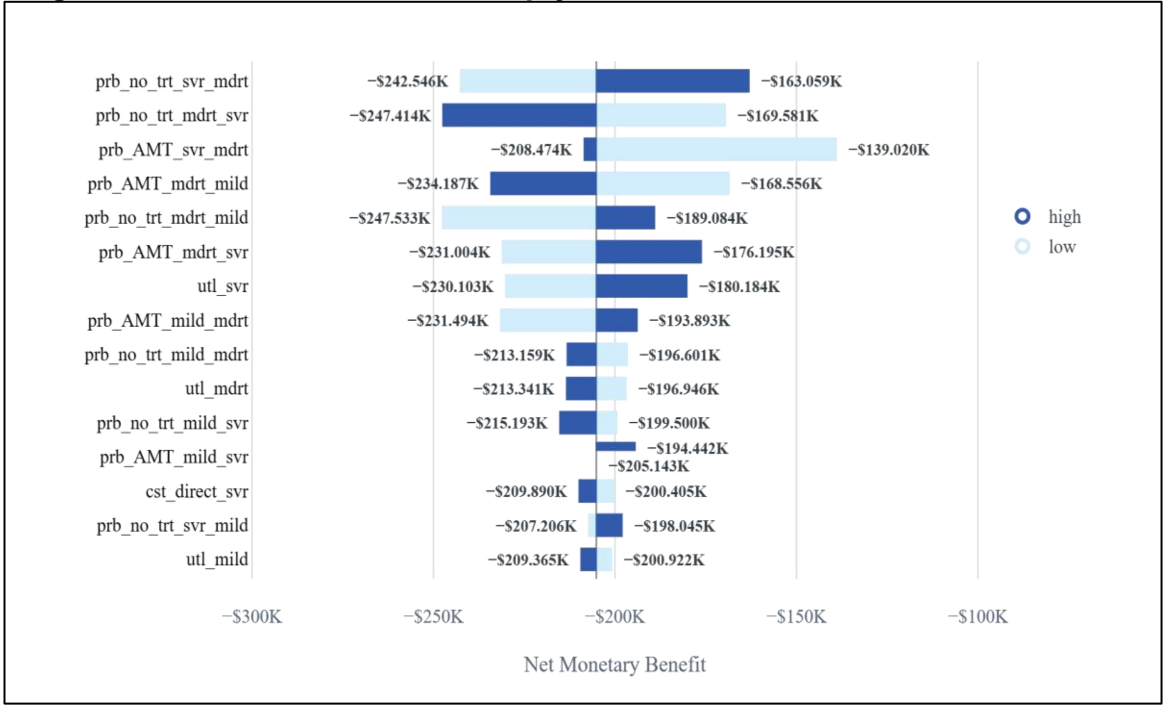

Abbreviations: AMT, Amitriptyline; no_trt, no treatment; mild_mdrt, mild to moderate; mild_svr, mild to severe; prb, probabillity; mdrt_mild, moderate to mild; mdrt_svr, moderate to severe; svr_mild, severe to mild; svr_mdrt, severe to moderate; utl_mdrt, utility moderate; utl_svr, utility severe; cst_direct_svr, direct cost (severe state).

© 2026 Downen SS et al. *JAMA Network Open*.

eFigure 3. Tornado Diagram of Deterministic Sensitivity Analyses, US Societal Perspective

eFigure 3A. Duloxetine 60mg vs. Amitriptyline

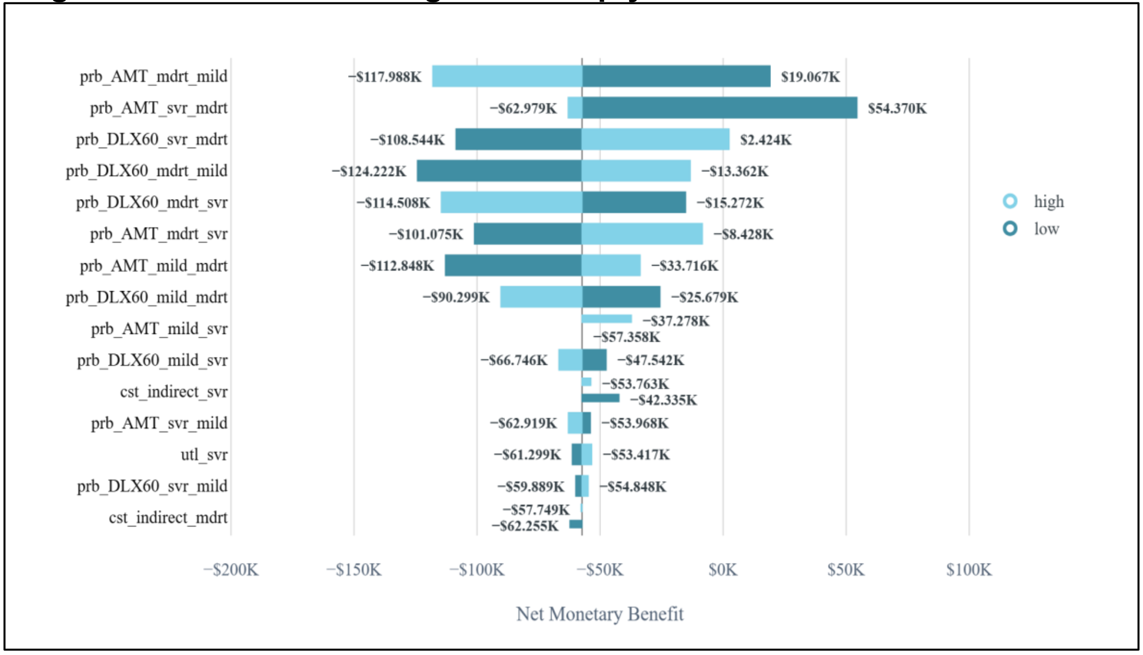

Abbreviations: AMT, Amitriptyline; DLX_60, Duloxetine 60mg; mild_mdrt, mild to moderate; mild_svr, mild to severe; prb, probabillity; mdrt_mild, moderate to mild; mdrt_svr, moderate to severe; svr_mild, severe to mild; svr_mdrt, severe to moderate; utl_mdrt, utility moderate; utl_svr, utility severe; cst_direct_svr, direct cost (severe state).

eFigure 3B. Duloxetine 120mg vs. Amitriptyline

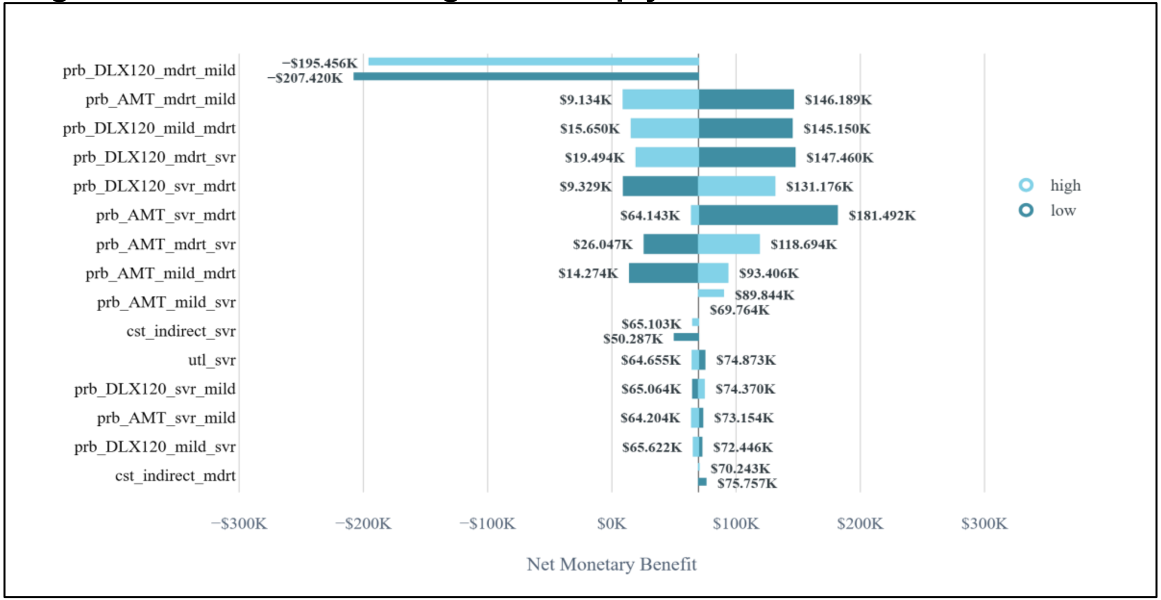

Abbreviations: AMT, Amitriptyline; DLX_120, Duloxetine 120mg; mild_mdrt, mild to moderate; mild_svr, mild to severe; prb, probabillity; mdrt_mild, moderate to mild; mdrt_svr, moderate to severe; svr_mild, severe to mild; svr_mdrt, severe to moderate; utl_mdrt, utility moderate; utl_svr, utility severe; cst_direct_svr, direct cost (severe state).

eFigure 3C. Milnacipran 100mg vs. Amitriptyline

© 2026 Downen SS et al. *JAMA Network Open*.

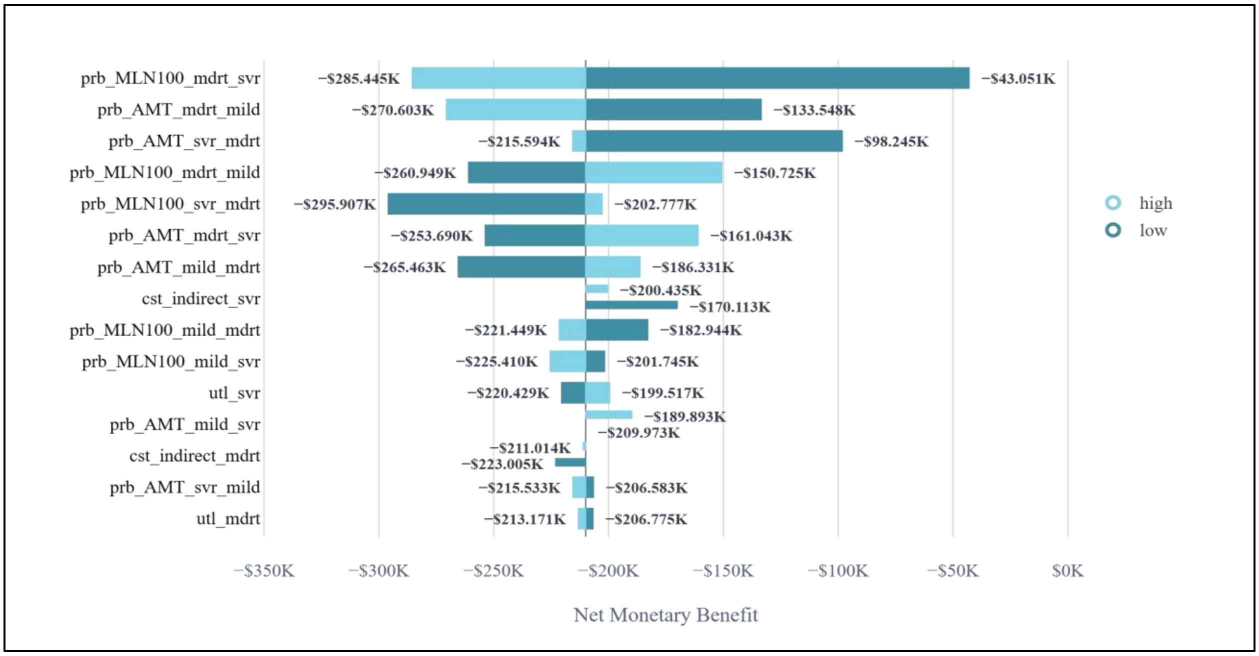

Abbreviations: AMT, Amitriptyline; MLN_100, Milnacipran 100mg; mild_mdrt, mild to moderate; mild_svr, mild to severe; prb, probabillity; mdrt_mild, moderate to mild; mdrt_svr, moderate to severe; svr_mild, severe to mild; svr_mdrt, severe to moderate; utl_mdrt, utility moderate; utl_svr, utility severe; cst_direct_svr, direct cost (severe state).

eFigure 3D. Milnacipran 200mg vs. Amitriptyline

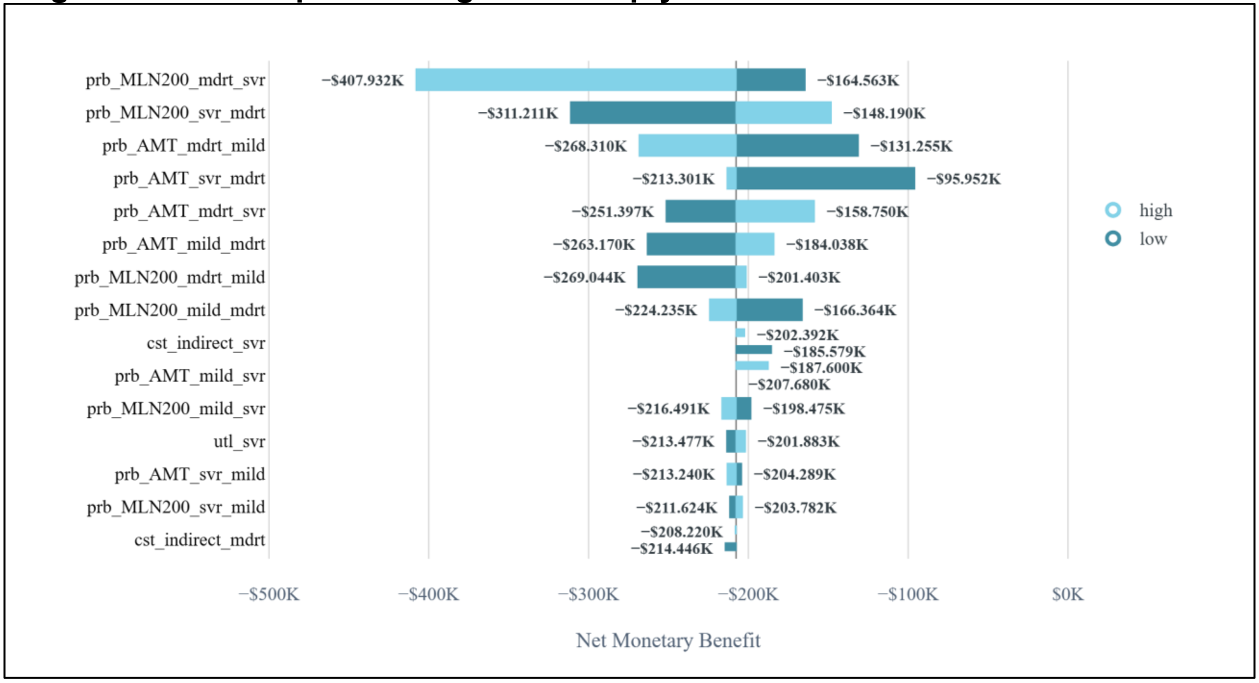

Abbreviations: AMT, Amitriptyline; MLN200, Milnacipran 200mg; mild_mdrt, mild to moderate; mild_svr, mild to severe; prb, probabillity; mdrt_mild, moderate to mild; mdrt_svr, moderate to severe; svr_mild, severe to mild; svr_mdrt, severe to moderate; utl_mdrt, utility moderate; utl_svr, utility severe; cst_direct_svr, direct cost (severe state).

© 2026 Downen SS et al. *JAMA Network Open*.

eFigure 3E. Pregabalin 150mg vs. Amitriptyline

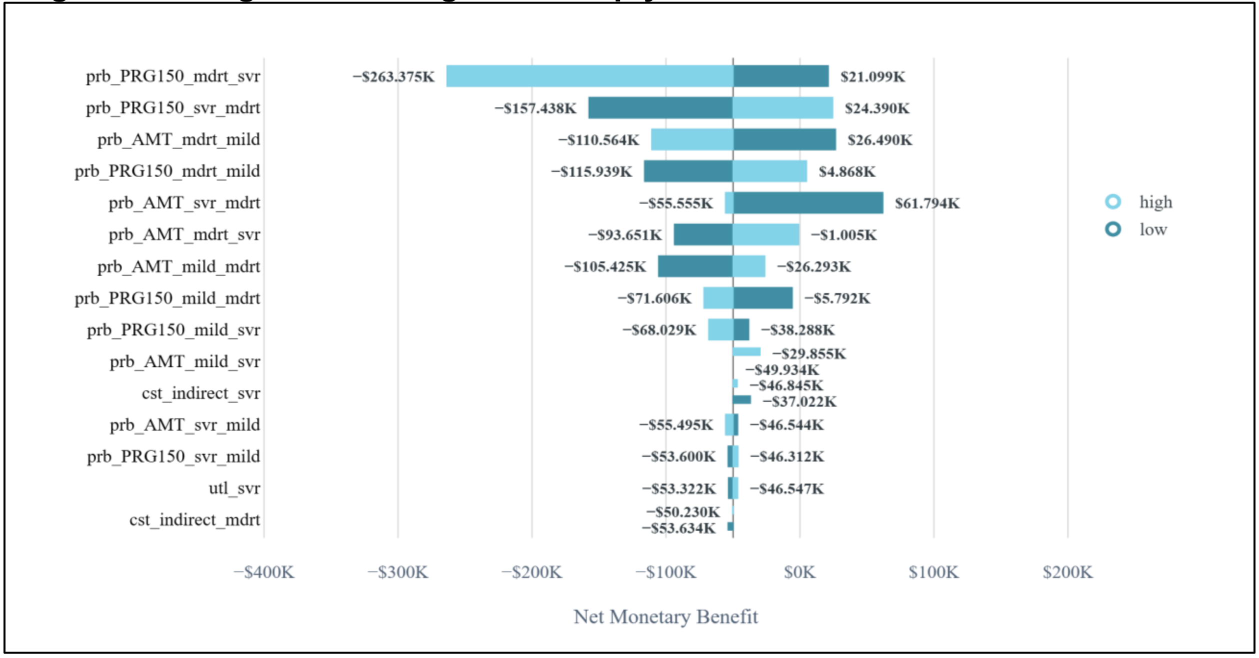

Abbreviations: AMT, Amitriptyline; PRG_150, Pregabalin 150mg; mild_mdrt, mild to moderate; mild_svr, mild to severe; prb, probabillity; mdrt_mild, moderate to mild; mdrt_svr, moderate to severe; svr_mild, severe to mild; svr_mdrt, severe to moderate; utl_mdrt, utility moderate; utl_svr, utility severe; cst_direct_svr, direct cost (severe state).

eFigure 3F. Pregabalin 300mg vs. Amitriptyline

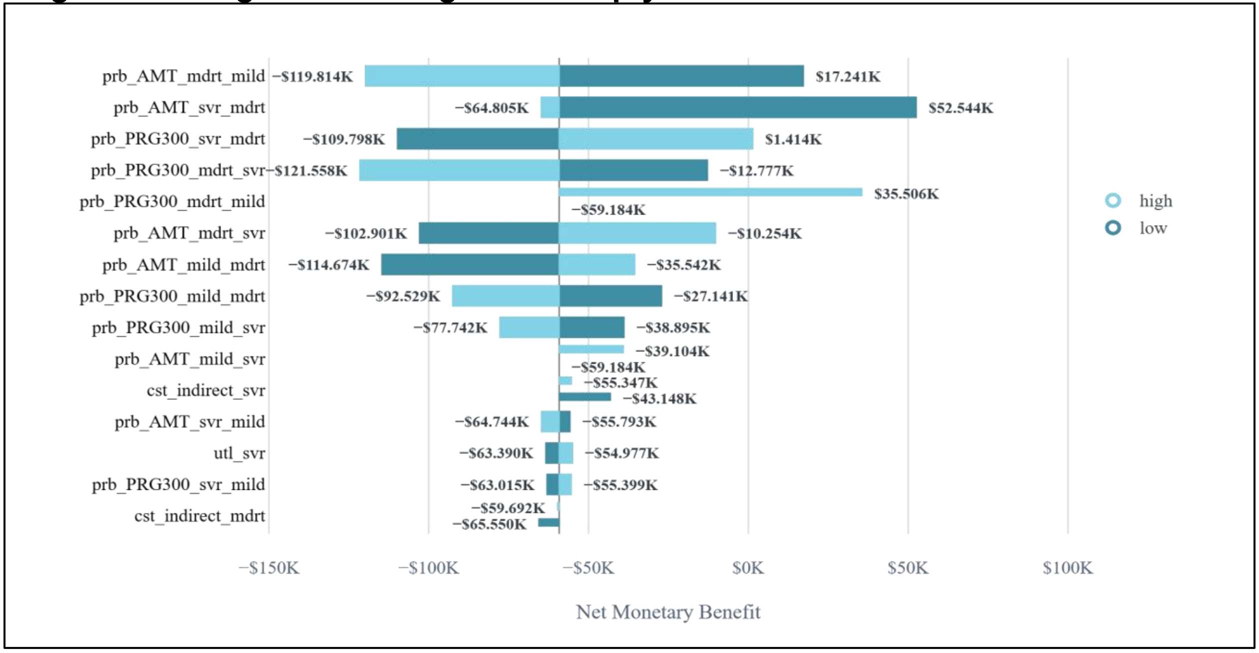

Abbreviations: AMT, Amitriptyline; PRG_300, Pregabalin 300mg; mild_mdrt, mild to moderate; mild_svr, mild to severe; prb, probabillity; mdrt_mild, moderate to mild; mdrt_svr, moderate to severe; svr_mild, severe to mild; svr_mdrt, severe to moderate; utl_mdrt, utility moderate; utl_svr, utility severe; cst_direct_svr, direct cost (severe state).

© 2026 Downen SS et al. *JAMA Network Open*.

eFigure 3G. Pregabalin 450mg vs. Amitriptyline

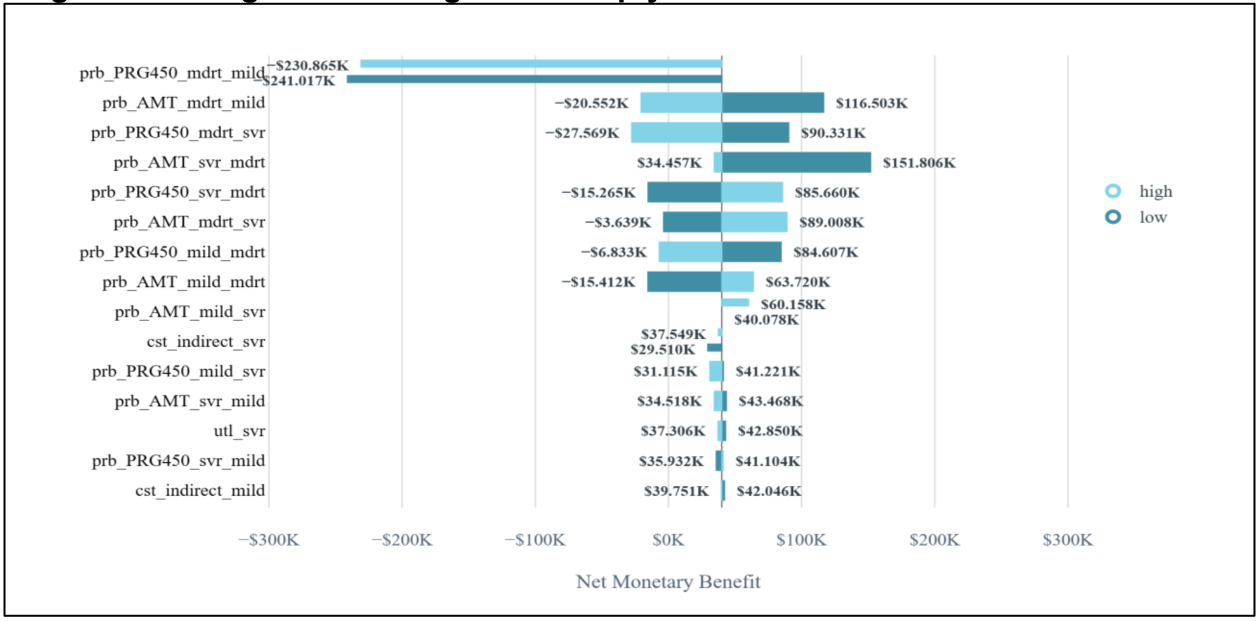

Abbreviations: AMT, Amitriptyline; PRG_450, Pregabalin 450mg; mild_mdrt, mild to moderate; mild_svr, mild to severe; prb, probabillity; mdrt_mild, moderate to mild; mdrt_svr, moderate to severe; svr_mild, severe to mild; svr_mdrt, severe to moderate; utl_mdrt, utility moderate; utl_svr, utility severe; cst_direct_svr, direct cost (severe state).

eFigure 3H. Pregabalin 600mg vs. Amitriptyline

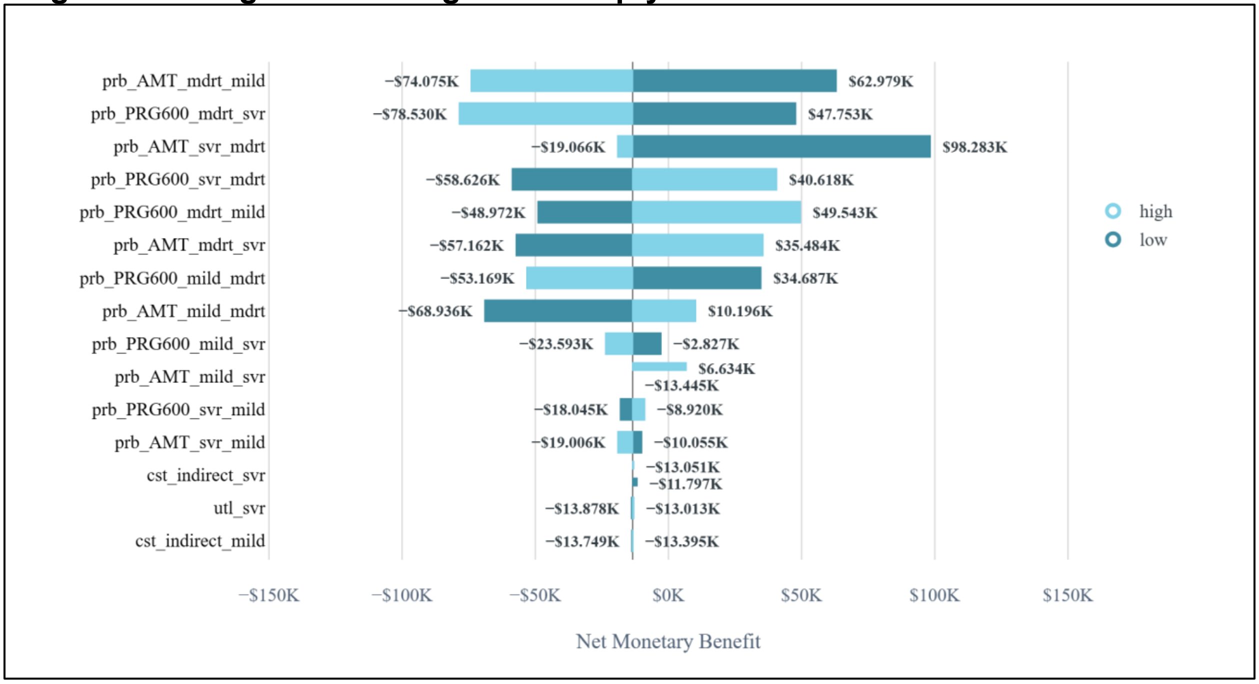

Abbreviations: AMT, Amitriptyline; PRG_600, Pregabalin 600mg; mild_mdrt, mild to moderate; mild_svr, mild to severe; prb, probabillity; mdrt_mild, moderate to mild; mdrt_svr, moderate to severe; svr_mild, severe to mild; svr_mdrt, severe to moderate; utl_mdrt, utility moderate; utl_svr, utility severe; cst_direct_svr, direct cost (severe state).

© 2026 Downen SS et al. *JAMA Network Open*.

eFigure 3I. No treatment vs. Amitriptyline

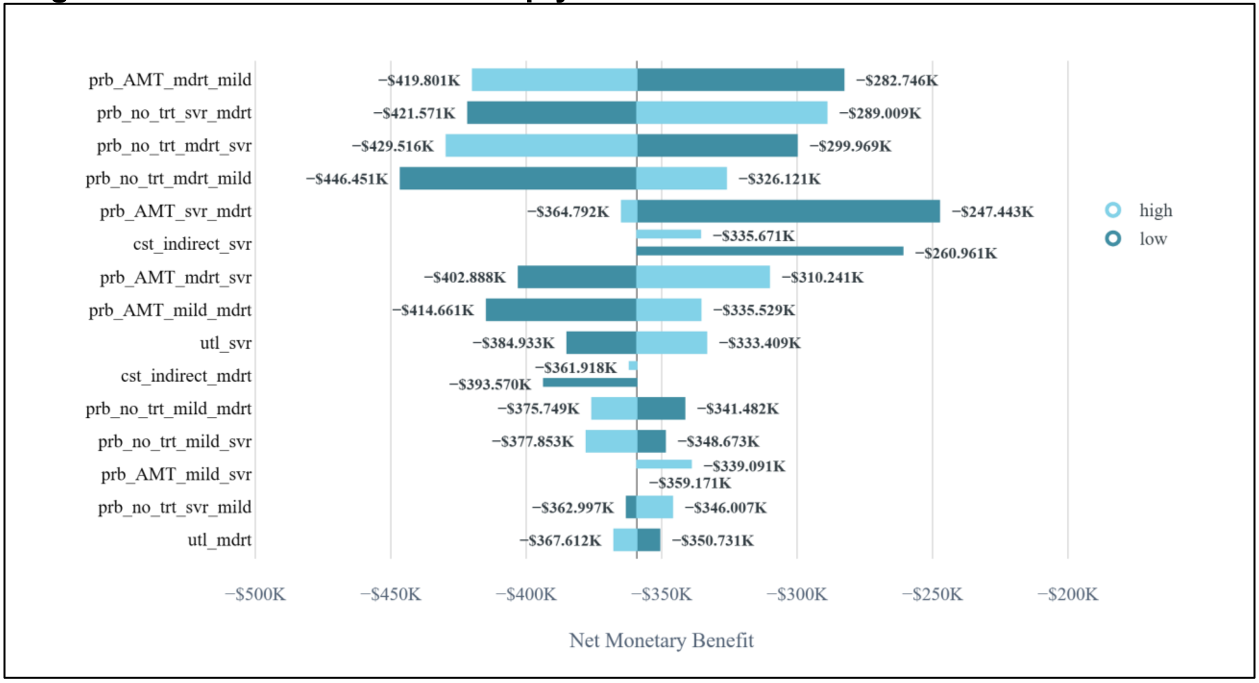

Abbreviations: AMT, Amitriptyline; no_trt, no treatment; mild_mdrt, mild to moderate; mild_svr, mild to severe; prb, probabillity; mdrt_mild, moderate to mild; mdrt_svr, moderate to severe; svr_mild, severe to mild; svr_mdrt, severe to moderate; utl_mdrt, utility moderate; utl_svr, utility severe; cst_direct_svr, direct cost (severe state).

© 2026 Downen SS et al. *JAMA Network Open*.

eReferences

1. Schaefer C, Chandran A, Hufstader M, et al. The comparative burden of mild, moderate and severe fibromyalgia: results from a cross-sectional survey in the United States. *Health Qual Life Outcomes.* 2011;9:71. Doi:10.1186/1477-7525-9-71.
2. Chandran A, Schaefer C, Ryan K, Baik R, McNett M, Zlateva G. The comparative economic burden of mild, moderate, and severe fibromyalgia: results from a retrospective chart review and cross-sectional survey of working-age U.S. adults. *J Manag Care Pharm.* 2012;18(6):415-426. Doi: 10.18553/jmcp.2012.18.6.415.
3. Red Book Pharmacy's Fundmental Reference. Montvale, NJ: Thomson Healthcare; 2009
4. Arias E, Xu JQ, Tejada-Vera B, Bastian B. US state life tables, 2021. Natl Vital Stat Rep. 2024;73(7). doi:10.15620/cdc/157499.
5. Husereau D, Drummond M, Augustovski F, et al. Consolidated Health Economic Evaluation Reporting Standards 2022 (CHEERS 2022) Statement: Updated Reporting Guidance for Health Economic Evaluations. *Value Health.* 2022;25(1):3-9. doi:10.1016/j.jval.2021.11.1351.

© 2026 Downen SS et al. *JAMA Network Open*.
