# Supplementary material for: Cost-Effectiveness of Pregabalin, Duloxetine, and Milnacipran vs Amitriptyline for Moderate to Severe Fibromyalgia
Source: JAMA Netw Open. 2026 Feb 3;9(2):e2557536. doi: 10.1001/jamanetworkopen.2025.57536 (PMC12869342; doi:10.1001/jamanetworkopen.2025.57536)
Supplement: Supplement 2. — Data Sharing Statement [file jamanetwopen-e2557536-s002.pdf]

Data Sharing Statement

Downen. Cost-Effectiveness of Pregabalin, Duloxetine, and Milnacipran Compared With Amitriptyline in Moderate-to-Severe Fibromyalgia. *JAMA Netw Open*. Published February 03, 2026. doi:10.1001/jamanetworkopen.2025.57536

Data
Data available: Yes
Data types: Data (not involving human participants)
How to access data: This is a simulation model. Input data is reported in the manuscript and supplementary files
When available: With publication

Supporting Documents
Document types: None

Additional Information
Who can access the data: Anyone
Types of analyses: This is a simulation model. Input data is reported in the manuscript and supplementary files
Mechanisms of data availability: Email
